# Synthesis, Spectroscopy, Light Stability, Single-Crystal Analysis, and In Vitro Cytotoxic Activity on HepG2 Liver Cancer of Two Novel Silver(I) Complexes of Miconazole

**DOI:** 10.3390/ijms21103629

**Published:** 2020-05-21

**Authors:** Karolina Stryjska, Lidia Radko, Lilianna Chęcińska, Joachim Kusz, Andrzej Posyniak, Justyn Ochocki

**Affiliations:** 1 Department of Bioinorganic Chemistry, Chair of Medicinal Chemistry, Medical University of Lodz, Muszyńskiego 1, 90-151 Łódź, Poland; karolina.stryjska@umed.lodz.pl; 2 Department of Pharmacology and Toxicology, National Veterinary Research Institute, Al. Partyzantów 57, 24-100 Puławy, Poland; lidia@piwet.pulawy.pl (L.R.); aposyn@piwet.pulawy.pl (A.P.); 3 Faculty of Chemistry, University of Lodz, Pomorska 163/165, 90-236 Łódź, Poland; lilianna.checinska@chemia.uni.lodz.pl; 4 Institute of Physics, University of Silesia, 75 Pułku Piechoty 1, 41-500 Chorzów, Poland; joachim.kusz@us.edu.pl

**Keywords:** silver(I) complex, cytotoxicity, NMR spectroscopy, IR spectroscopy, X-ray crystallography, Balb/c 3T3 cell line, HepG2 cell line

## Abstract

Two novel silver(I) complexes of the biologically active ligand miconazole in the form of Ag(MCZ)_2_X (MCZ = 1-[2-(2,4-dichlorobenzyloxy)-2-(2,4-dichlorophenyl)ethyl]-1*H*-imidazole]; X = NO_3_^−^ (**1**), ClO_4_^−^ (**2**)) were synthesized and fully characterized. The complexes were obtained by reactions of Ag(I) salts with miconazole (MCZ). Silver(I) complexes were characterized by elemental analysis, ^1^H-NMR and infrared (IR) spectroscopy, electrospray ionization (ESI)-MS spectrometry, and X-ray-crystallography. This work also presents a cytotoxicity study of the silver(I) complexes of miconazole and appropriate silver(I) salts using Balb/c 3T3 and HepG2 cell lines. The cytotoxicity of the compounds was assessed based on four biochemical endpoints: lysosomal activity (neutral red uptake (NRU) assay), mitochondrial activity (3-(4,5-dimethyl-2-thiazolyl)-2,5-diphenyl-2-H-tetrazolium bromide (MTT) assay), total protein content (TPC assay), and cellular membrane integrity (lactate dehydrogenase (LDH) assay). The cancer HepG2 cells were more sensitive to the complexes tested, and the most affected endpoint was cellular membrane damage compared to Balb/c 3T3 fibroblasts. Moreover, study complexes inhibited the growth of cancer cells at submicromolecular concentrations (0.26–0.47 μM) lower than that required for the anticancer agent, cisplatin, in MTT, NRU, and TPC assays. Both complexes were characterized by higher toxicity to human cancer cells (HepG2) than silver(I) salts and the free ligand. Combination of Ag(I) salts with miconazole is associated with the marked improvement of cytotoxic activities that can be considered as the significant point in the construction of a new generation of antineoplastic agents.

## 1. Introduction

Chemotherapeutic agents play a significant role in medicine and veterinary services. Consequently, many studies aimed at discovering new forms of these agents are being conducted today. The discovery of new metal complexes, which have innovative therapeutic effects in medical practice, is of great interest to researchers [1,2]. Moreover, many of them focus on the synthesis of new complexes of transition metals that show anticancer properties against various cancer cells [3,4,5,6,7,8,9,10], and they can be an alternative to chemotherapy that is associated with severe side effects [11].

Cisplatin (cis-diamminedichloroplatinum(II)) is a medicine used against various types of human cancers such as lung, ovarian, head, bladder, and testicular cancer. However, due to numerous side effects such as kidney problems, allergic reactions, the development of resistance in cancer cells, or hemorrhage, its use is limited [12,13].

Silver and its compounds are effective and widely used antimicrobial agents [14,15,16]. Silver compounds were used to prevent infections before antibiotics were invented. For this purpose, silver nitrate solution was most often used, followed by cream containing sulfadiazine silver(I) salts, which was mainly used for burns. Silver-based compounds are also used in medical devices such as catheters, implants, and surgical sutures [17,18].

It is proven that many silver complexes showed better biological activity than the free ligand, which is the basis for their use in medical purposes [19,20,21,22].

Silver(I) complexes have a strong antibacterial and antifungal effect, characterized by low toxicity to humans [23,24,25,26,27]. Recent studies show that silver complexes also have strong anticancer properties [6,28] and are cytotoxic to various cell types [29,30].

Currently, toxicity testing is a necessary stage in preclinical testing of a potential therapeutic agent [31,32,33].

In vivo research is problematic due to related ethical and social issues, as well as legal and economic concerns. For this reason, in vitro tests are used in many cases to limit animal experimentation. Cytotoxicity studies may provide reliable and useful information on the potential mechanism of action due to the fact that the impairment of basic cellular functions results in toxic effects within the whole organism [34,35,36,37,38,39].

The Organization for Economic Cooperation and Development (OECD) recommends a Balb/c 3T3 cell line for testing the overall toxicity of chemicals [36]. In the case of cancer patients, HepG2 cells are widely used as a test system to predict toxicity. They are used as target cells in the evaluation of liver cancer toxicity in humans [40].

**Figure 1:**
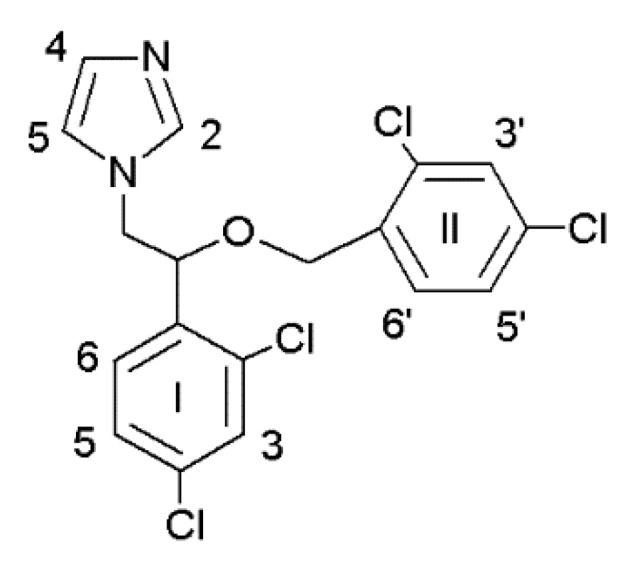
Chemical structure of miconazole.

Miconazole (MCZ) is a drug used in medicine with antifungal activity [41,42,43,44]. The chemical structure of miconazole is presented in Figure 1. It is a white powder, soluble in ethanol, hardly soluble in methanol, and practically insoluble in water. It is used to treat systemic fungal infections such as candidiasis and to treat skin infections such as dandruff. Miconazole eliminates itching of the skin, which often accompanies infections caused by yeast and dermatophytes. In addition, the drug has antibacterial activity against Gram-positive bacteria. It is used in the form of a lotion, cream, powder, chewing gum, or buccal tablets in the case of oropharyngeal candidiasis [45]. A commonly used drug with miconazole nitrate (20 mg/1 g) as the active substance is Daktarin^®^ [46].

The antifungal effect of miconazole is to inhibit the biosynthesis of ergosterol in the cell membrane of microorganisms. At low concentrations, miconazole affects the cytochrome P450 system in fungal cells, resulting in inhibition of 14-α-demethylation of the ergosterol biosynthesis stage [47].

The subject of many studies was the synergistic analysis of coordination compounds of transition metals, including silver, with imidazole derivatives. The obtained results showed that such compounds have promising therapeutic potential and can be used as antifungal, antibacterial, and anticancer agents [48]. The synthesis and characterization of silver complexes with benzimidazole derivatives were also described. The results showed that these compounds have stronger antibacterial and anticancer properties than the free ligand. In addition, these compounds are not toxic to healthy 10T1/2 cells (murine fibroblasts) [49].

The goal of present study was to develop a fast and simple one-step procedure for the direct synthesis of new complexes using miconazole, the antifungal synthetic derivative of imidazole which is used in the treatment of candida skin infections, and silver nitrate, which is used as an anti-infective agent. The method describes the use of silver(I) nitrate or silver(I) perchlorate and miconazole as molecules directly forming final complexes.

The compounds were characterized by spectroscopic methods (^1^H-NMR, infrared (IR)), elemental analysis, electrospray ionization (ESI)-MS, and X-ray measurements. Additionally, Quantum Theory of Atoms in Molecules (QTAIM) [50] was employed to analyze two types of chemical bonds: silver–nitrogen between the silver cation and nitrogen from azole ligands, and silver–oxygen (or fluorine) between silver(I) and counter anions (NO_3_^−^, ClO_4_^−^, CF_3_COO^−^, CH_3_SO_3_^−^, BF_4_^−^).

The aim of the research was also to determine anticancer activity against human hepatocellular carcinoma. The cytotoxic potential of the compounds was tested against the HepG2 human liver cancer cell line and murine fibroblast Balb/c3T3 cell line compared to cisplatin, by calculating the half maximal inhibitory concentration (IC_50_) for the compounds tested. Indeed, the goal was achieved, and the results are presented below.

## 2. Results and Discussion

### 2.1. Synthesis of the Compounds [Ag(MCZ)_2_NO_3_] *(**1**)* and [Ag(MCZ)_2_ClO_4_] (***2**)*

**Scheme 1:**
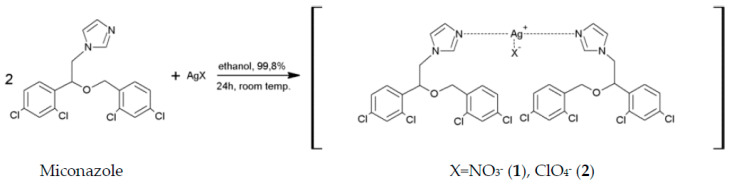
Synthesis of silver(I) complexes of miconazole.

A complex of miconazole with appropriate silver(I) salts was obtained by reacting the AgX [X = NO_3_^−^, ClO_4_^−^] with miconazole (1:2) (Scheme 1) in ethanol. The resulting compound was obtained with good yield and purity.

### 2.2. NMR Spectroscopy

Complexes and free drug spectra were recorded in CDCl_3_ as the solvent. In the free ligand spectrum, doublets appeared at 7.26 and 7.27 ppm, which can be attributed to the H4 and H5 protons of the imidazole ring. Unfortunately, there are no H2-derived signals in the imidazole ring in the CDCl_3_ solvent. The signals at 7.38 and 7.48 ppm from H3 and H3′ protons appeared on the spectrum. Signals at 7.32 and 7.33 ppm are also observed and are attributed to H5′ and H6′ protons, while signals at 7.20 and 7.05 ppm are attributed to H5 and H6 protons. The signal at 4.10 ppm can be assigned to aliphatic CH proton, and signals between 4.08–4.52 ppm may be attributed to two aliphatic CH_2_ protons [51].

The spectra of silver(I) complexes show slight shifts to a higher field compared to the free MCZ ligand (Table 1). According to the literature data, slight shifts seem to be typical for silver(I) complexes [25,52]. In addition, compared to the ligand, the spectra of the complex show signals at 8.08 ppm for compound **1** and 8.20 ppm for compound **2**, which is attributed to H2 in the imidazole ring. The ^1^H-NMR spectra of the compounds are presented in the (Supplementary Materials Figure S1).

### 2.3. IR Spectroscopy

Infrared spectroscopy was used to confirm the formation of a coordination bond between the metal and the organic ligand and to confirm the changes occurring as a result of interaction of miconazole with silver(I) salts.

In the free ligand spectrum, the bands at 1589 and 1562 cm^−1^ are attributed to C–C stretching vibrations of the two dichlorobenzene groups. The band at 1505 cm^−1^ corresponds to C–C stretching vibrations of the imidazole group and the CH bending of the imidazole group, i.e., the C6 aliphatic part of miconazole. The band at 1468 cm^−1^ is attributed to CH bending vibrations of two dichlorobenzene groups and CH bending of the C6 and C17 [53]. In addition, in the miconazole spectrum, a band at 3151 cm^−1^ is observed, which is attributed to the CN vibration of the imidazole group, with the band at 3109 cm^−1^ corresponding to aromatic CH stretching, and those at 1380 cm^−1^ and 1334 cm^−1^ corresponding to C–H bending and C–N stretching [54].

The silver(I) spectra of the complexes (Supplementary Materials Figure S2) contain characteristic miconazole vibration bands. These are the C–C stretching vibration bands of two dichlorobenzene groups that occurr in the IR spectra of the complexes at 1588 (**1**) and 1590 cm^−1^ (**2)**, as well as at 1562 (**2**) and 1563 cm^−1^ (**1**). The band corresponding to the CH bending vibrations of the two dichlorobenzene groups is located in the spectra of the complexes at 1470 (**2**) and 1471 cm^−1^ (**1**), respectively. The band at 1092 cm^−1^ on the spectrum of the free drug corresponding to the stretching vibrations of the C–O–C group is found in the obtained coordination compounds at 1095 cm^−1^ for compound **1** and at 1098 cm^−1^ for compound **2**. This band remains intact in solid complexes, indicating its noninvolvement in metal ion coordination. The bands at 1722 cm^−1^ for **1** and 1727 cm^−1^ for **2** appear on the spectra of the complexes. This band is not present in the spectrum of the free ligand. Due to the lack of literature, it can be assumed that these bands come from the C=N group of stretching vibrations of the imidazole nitrogen. The presence of these bands in comparison to the miconazole spectrum indicates the coordination of the drug with silver(I) ions by the imidazole group nitrogen atom, which is confirmed by the X-ray structural studies of the obtained single crystals of the complexes. In addition, all peaks recorded on the spectra of the complexes are more intense compared to the free drug.

### 2.4. Light Stability of the Compounds

The light stability of the [Ag(MCZ)_2_NO_3_] and [Ag(MCZ)_2_ClO_4_] complexes and silver(I) salts (AgNO_3_, AgClO_4_) was studied in normal daylight in air atmosphere and in the dark [20,55]. The 0.05 mol/L solutions of the compounds were applied onto the following backgrounds: tissue paper, paper, glass, and synthetic leather. Stability was monitored visually for 108 h, making image documentation after 0, 1, 4, 18, 24, 40, 48, 52, 60, 84, and 108 h.

Appropriate silver(I) salts darkened on tissue paper and paper after 1 h of exposure to light. The complexes remained unchanged until 24 h. The salts of silver(I) completely darkened after 24 h on all backgrounds. The decomposition of the complex compounds was mild and slightly darkened after three days. The complexes decomposed most quickly on the leather imitation. On a glass and tissue paper background, the compounds remained unchanged. A slight darkening of the silver–miconazole complexes was observed on the paper. 

The complexes obtained clearly showed better stability during exposure to light than silver(I) salts. The experiment which was conducted simultaneously without access to light proceeded as expected. The complex compounds showed better stability than Ag(I) salts, whereby their decomposition progressed slower than in the case of exposure to light. Light significantly influences the rate of silver nitrate decomposition.

The experiment showed that the compounds studied could potentially be used in pharmacy as an external agent in the form of ointments or gels (Supplementary Materials Figure S3).

### 2.5. X-ray Single-Crystal Structures

Supplementary crystallographic data for this paper can be found at the Cambridge Crystallographic Data Center under the depository numbers CCDC 1977953 (**1**) and 1977954 (**2**).

**Figure 2:**
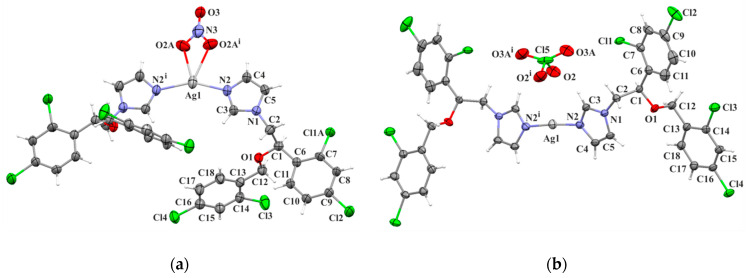
Molecular structures of Ag(I) complexes with miconazole and nitrate (**1**) (**a**) and perchlorate (**2**) (**b**) counter ions. The ellipsoids are drawn at the 30% (**1**) and 50% (**2**) probability level. The B-parts of the disordered atoms are omitted for clarity. Symmetry codes: for **1** (i) 1 − *x*, *y*, ½ − *z*; for **2** (i) 1 − *x*, *y*, ½ − *z*.

The molecular structures of silver(I) complexes of miconazole and two different counter ions, nitrate (**1**) and perchlorate (**2**), are shown in Figure 2. The crystal data are summarized in Table 2. Both complexes crystallize in a monoclinic system, in the *C*2/c space group, *Z* = 4. In both structures, Ag(I) lies on a two-fold axis and is coordinated by two nitrogen atoms (N2) from symmetrically related miconazole ligands. Table 3 lists the bond lengths and angles around the silver atom. The silver donor bond lengths differ significantly. Moreover, the spatial arrangement of miconazole ligands around the metal atom is also different. The N–Ag–N bond angle is close to be linear in **2** in contrast to strong bending observed in **1**. Furthermore, the two imidazole rings are almost coplanar in **1**, and the dihedral angle between them is 3.0(3)°, whereas, in **2**, the five-membered heterocyclic rings are inclined at the angle of 22.7(2)°. However, the most pronounced difference concerns the interaction between the molecular Ag(I) cation and counter ions. In complex **1,** the nitrate anion binds strongly to the silver center, whereas, in complex **2**, the perchlorate anion interacts very weakly.

**Figure 3:**
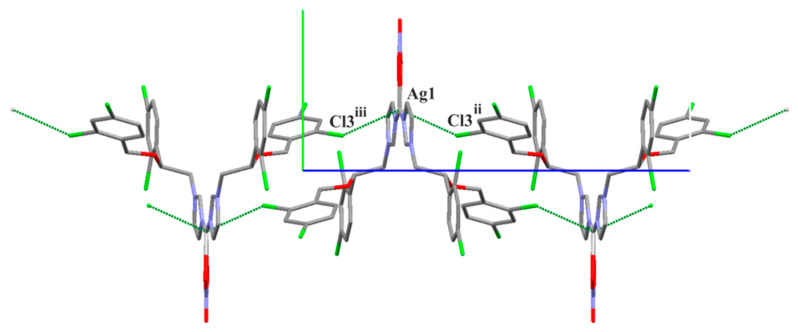
A part of the crystal structure of **1** showing close contacts of Ag⋯Cl (green dotted). A view down the crystallographic *a* axis. Hydrogen atoms are omitted for clarity. Symmetry codes: (ii) 1 − *x*, −*y*, 1 − *z*; (iii) *x*, −*y*, −½ + *z*.

**Figure 4:**
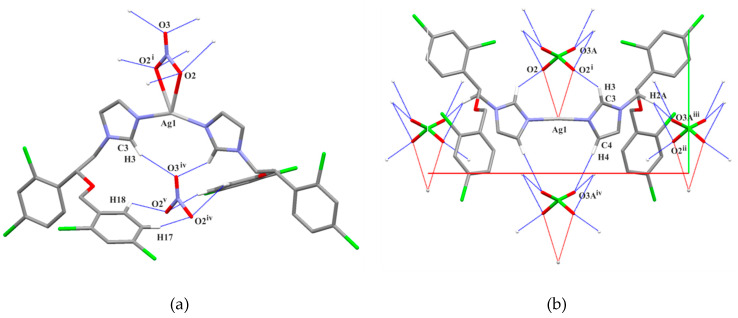
A scheme C–H⋯O of hydrogen bonds (blue dotted) between cations and anions of **1** (**a**) and **2** (**b**). Red dotted show weak Ag⋯O contacts between cations and anions of **2** (**b**). In both cases, hydrogen atoms not involved in H-bonds are omitted for clarity. Symmetry codes for **1**: (i) 1 − *x*, *y*, ½ − *z*; (iv) *x*, −1 + *y*, *z*; (v) 1 − *x*,−1 + *y*, ½ − *z*. Symmetry codes for **2**: (i) 1 − *x*, *y*, ½ − *z*; (ii) −½ + *x*, −½ + *y*, *z*; (iii) *x*, −*y*, −½ + *z*; (iv) 1 − *x*, −1 + *y*, ½ − *z*.

The analysis of close contacts in the structure **1** revealed the Ag⋯Cl intermolecular interactions which complement the coordination of Ag(I). Figure 3 demonstrates the molecular chains formed by a propagation of Ag⋯Cl contacts along the crystallographic *c* axis. These chains are additionally stabilized by the C15–H15⋯π (heterocyclic ring*)* intermolecular interaction. The geometries of intermolecular interactions are listed in Table 3. The C–H⋯O hydrogen bonds link the miconazole ligands with nitrate anions along the *b* axis (Figure 4a). Finally, molecular layers *bc* are observed. There are aromatic π⋯π stacking interactions of *Cg*1⋯*Cg*2 (Supplementary Materials Table S1) between adjacent layers; however, π-electrons of the dichlorophenyl rings C13–C18 (*Cg*3⋯*Cg*3) interact with each other within the layer.

As far as structure **2** is concerned, there are no additional close contacts with the silver center. Similarly to structure **1**, C–H⋯O hydrogen bonds are observed between the miconazole ligands and perchlorate anions (Table 4, Figure 4b). The propagation of weak hydrogen bonds leads to the formation of molecular *ab* layers (Supplementary Materials Figure S4). The short contacts of Cl3⋯Cl3 (−*x*, 1 − *y*, 1 − *z*) are observed between these layers. Aromatic π⋯π interactions are also found (Table S1, Supplementary Materials) for Cg3⋯Cg2 within the layer and Cg3⋯Cg3 between the adjacent layers.

### 2.6. QTAIM Analysis

Two types of chemical bonds were analyzed: Ag–N between the silver cation and nitrogen from azole ligands, and Ag–O (or F) between silver(I) and counter anions (NO_3_^−^, ClO_4_^−^, CF_3_COO^−^, CH_3_SO_3_^−^, BF_4_^−^).

The results in Table 5 are listed according to ascending bond length. It can be seen that the silver(I) complexes of metronidazole (MTZ = 1-(2-hydroxyethyl)-2-methyl-5-nitro-1*H*-imidazole) [20] have quite similar bond lengths and they are located in the middle of the table, whereas the miconazole complexes are found on the top and the bottom of the table. Silver–nitrogen distances range from 2.06Å to 2.18Å, both extreme values corresponding to the analyzed complexes of silver with the miconazole ligand.

As the length of the bonds (*d*) increases, the N–Ag–N angle decreases; thus, the linearly attached ligands tend to characterize short distances. The relatively small electron density values (*ρ*_bcp_) reflect geometric changes. The positive sign of the Laplacian (∇^2^*ρ*_bcp_) points toward an electrostatic donor interaction, which is in agreement with the quite high values of the degree of iconicity (*G/ρ*_bcp_). The degree of covalency (*H/ρ*_bcp_ ratio) also seems to be small. Ellipticity (*ε*) remains almost unchanged, close to 0. On the other hand, delocalization index values (*δ*) ranging from 0.66 to 0.55 indicat the quite high covalent contribution for Ag–N bonds. 

In the case of Ag–O_anion_ interactions, the geometric trends are inverse to Ag–N bonds. Here, the miconazole–silver(I) complex with nitrate is found at the top of Table 5, whereas the miconazole–silver(I) complex with perchlorate is at the bottom of Table 5. In the former structures, the anion is connected very weakly with the metal. All parameters clearly show the dominant ionic character of all Ag–O_anion_ interactions, where the covalent contribution is negligible. In most cases, the delocalization index is around 0.2. Interestingly, a reasonable sharing of the electron pair (δ) is observed in the case of CF_3_COO and CH_3_SO_3_ anions in complexes with metronidazole Ag(I) complexes. The miconazole Ag(I) complex with perchlorate anion (**2**) differs from the others. It exhibits the weakest interaction between cationic and anionic molecules; the electron density and its Laplacian at the bond critical point of this interaction are close to zero. The perchlorate anion in the Ag(I) complex structure with the miconazole ligand behavs similarly to the BF_4_^−^ counter ion in the metronidazole Ag(I) complex. In both cases, there is no sharing of the electron pair.

### 2.7. Cytotoxic Activity

**Figure 5:**
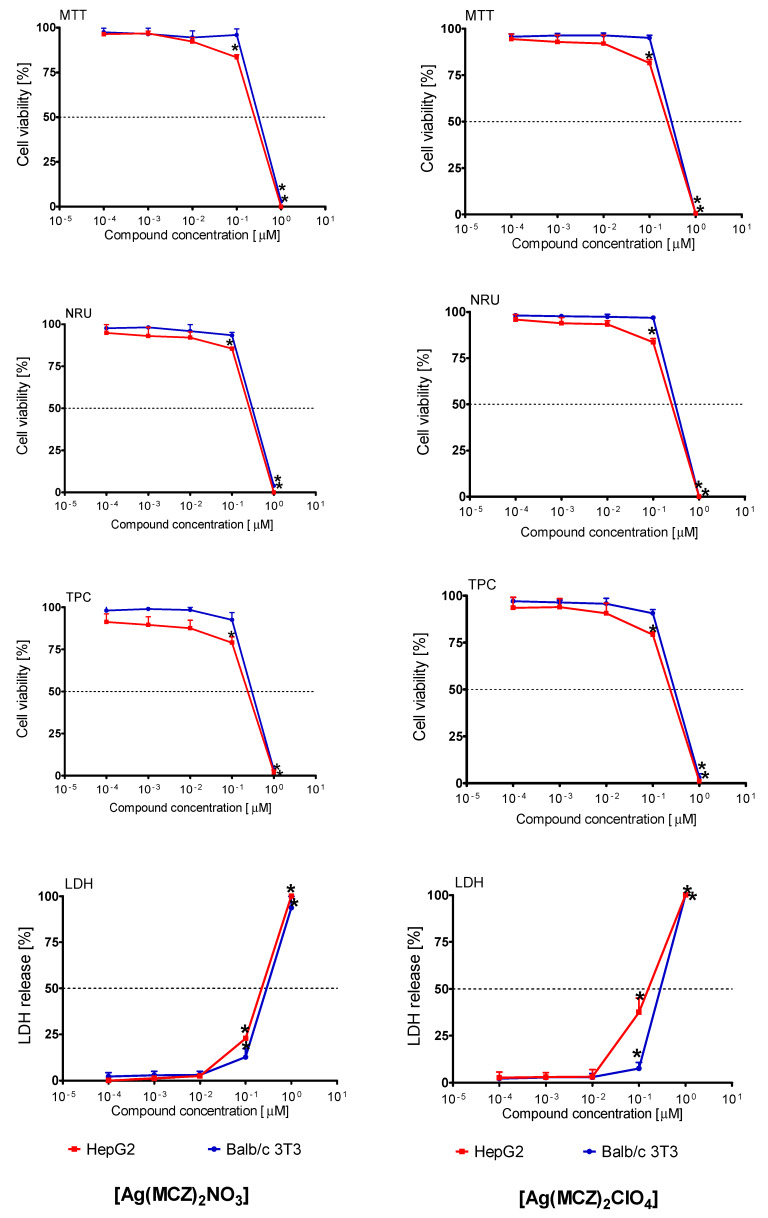
Concentration-dependent response curves for the studied compounds for HepG2 cells and Balb/c 3T3 fibroblasts measured by MTT, NRU, TPC, and LDH assays. The data are presented as means (*n* = 3) ± SD (* *p* ≤ 0.05).

The cytotoxic activity of the compounds was investigated in vitro against human hepatocellular carcinoma HepG2 cells and non-tumorigenic murine Balb/c 3T3 fibroblasts using four biochemical endpoints: mitochondrial (3-(4,5-dimethyl-2-thiazolyl)-2,5-diphenyl-2-H-tetrazolium bromide (MTT) assay) and lysosomal (neutral red uptake (NRU) assay) activity, total protein content (TPC assay), and cellular membrane integrity (lactate dehydrogenase (LDH) assay) after 72 h of exposition. Human HepG2 cells were more sensitive to the compounds than murine Balb/c 3T3 fibroblasts. The studied complexes with miconazole significantly inhibited all endpoints of the HepG2 cells starting with the 0.1 µM concentration (Figure 5). In the case of Balb/c 3T3 fibroblasts, at the higher concentration of 1.0 µM, the effects in MTT, NRU, and TPC assays were recorded (Figure 5). The study complexes in 0.1 µM concentration affected the cellular membrane (LDH assay), leading to disintegration of hepatoma cells and fibroblasts (Figure 5). The obtained cytotoxic concentrations (IC_20_ and IC_50_) values are shown in Table 6. The concentrations of the study complexes (IC_20_) necessary for 20% inhibition of hepatoma cell (HepG2) viability were about two-fold lower compared with values obtained for fibroblasts (Table 6). However, these values were higher compared to IC_20_ values for cisplatin on HepG2 cells (Table 6). The IC_50_ values for miconazole silver(I) complexes in all the assays used were ~0.5 μM in Balb/c 3T3 fibroblasts (Table 6). In the case of HepG2 cells, the IC_50_ values were lower compared to those obtained for fibroblasts as with the IC_20_ values. The lowest IC_50_ values were obtained for cancer cells in the LDH assay. It is worth noting that IC_50_ values in the LDH assay were lower for the [Ag(MCZ)_2_ClO_4_] complex (0.26 μM) compared with values for [Ag(MCZ)_2_NO_3_] (0.40 μM). However, these IC_50_ values were higher compared to cisplatin (0.01 μM). The IC_50_ values for cisplatin were higher than 1 µM in the MTT, NRU, and TPC assays for HepG2 cells and in all used assays for fibroblasts (Table 6). The studied silver(I) salts and MCZ were not toxic to the tested cell lines (Supplementary Materials Figure S5). The IC_50_ values were not calculated for these compounds in both cellular models (up to the highest concentration, 1 μM) (Table 6).

Recent evidence suggests that triazoles and miconazole exhibit antiproliferative effects, and they can be modified on their 1,2,4-triazole nucleus to generate different types of anticancer agents. Miconazole was also shown to be an effective anticancer drug. It was found to inhibit the proliferation of human cancer cells at a concentration up 10 μM, e.g., breast, bladder, and colorectal cancer cell lines, as well as acute myelogenous leukemia and osteosarcoma cells [56]. In both cases of the complexes [Ag(MCZ)_2_ClO_4_] and [Ag(MCZ)_2_NO_3_], the coordination of the metal to the ligand (MCZ) increased their cytotoxic activity, which was higher compared to the anticancer activity of cisplatin.

In our previous studies, we reported on the cytotoxicity of the compound [Ag(MTZ)_2_NO_3_] [57]. The study showed that non-tumorigenic murine Balb/c 3T3 fibroblasts were more sensitive to the test complex compared to HepG2 human liver cancer cells. On the other hand, the results in this paper for the compound [Ag(MCZ)_2_NO_3_] indicated the better efficacy of elimination of HepG2 cancer cells and lower toxicity to Balb/c 3T3 fibroblasts than the silver(I) complex of metronidazole. In addition, the [Ag(MCZ)_2_NO_3_] complex compared to [Ag(MTZ)_2_NO_3_] began to destroy HepG2 liver cancer cells at lower concentrations, silver(I) complexes of miconazole were up to tenfold more cytotoxic to cancer cells compared to the silver(I) complex of metronidazole [Ag(MTZ)_2_NO_3_] [57]. However, the model of action on cancer cells was the same for studied silver(I) complexes of imidazole derivatives, leading to the damage cell membrane. A comparison of both complexes suggests that the choice of ligand substantially contributed to the increased compound toxicity to tumor cells. In this case, the exchange of the ligand improved the anti-tumor properties.

## 3. Materials and Methods

### 3.1. Chemicals and Reagents

Miconazole (CAS: 22916-47-8; molecular weight 416.1 g/mol) was purchased from Alfa Aesar (Kandel, Germany). Silver nitrate (CAS: 7761-88-8; molecular weight: 169 g/mol), silver perchlorate (CAS: 7783-93-9; molecular weight: 207.3 g/mol), and cisplatin (CAS: 15663-27-1; molecular weight: 300 g/mol) were purchased from Sigma-Aldrich (Poznań, Poland). Triton X-100, trypan blue, dimethyl sulfoxide (DMSO), fetal bovine serum (FBS), bovine calf serum (BCS), neutral red dye (NR), Coomassie brilliant blue R-250 dye, 3-(4,5-dimethylthiazol-2-yl)-2,5-diphenyltetrazolium bromide (MTT), trypsin-ethylenedinitrilotetraacetic acid (EDTA), and antibiotic solution (10,000 U/mL of penicillin, 10 mg/mL of streptomycin) were purchased from Sigma–Aldrich Co. (Poznań, Poland).

All other chemicals were purchased from commercial suppliers and were of the highest available purity.

### 3.2. Synthetic Procedures

#### 3.2.1. Synthesis of [Ag(Miconazole)_2_NO_3_] (**1**)

Miconazole (MCZ) (1 mmol, 0.416 g) was dissolved in 10 mL of ethanol. AgNO_3_ solution (0.5 mmol, 0.085 g) in 10 mL of ethanol was added into the miconazole ethanolic solution and stirred for 24 h at room temperature. The white solid that was formed was filtered off and washed with portions of diethyl ether. The white crystals suitable for X-ray diffraction were grown from pure acetonitrile at room temperature. Molecular weight (M_W_) 1002.05 g/mol; yield: (0.40 g, 80%); melting point (m.p.) 153–155 °C. ESI-MS (methanol) *m*/*z*: 938.87 [Ag(MCZ)_2_]^+^. El. anal. Measured (calc.%): C—43.16 (43.15); H—2.47 (2.81); N—6.98 (6.99). ^1^H-NMR (600 MHz, CDCl_3_) δ (ppm): 4.13 (m, 4H, 2 × N—CH_2_), 4.16 (m, 2H, 2 × CH—O), 4.55 (s, 4H, 2 × CH_2_—O), 7.08 (s, 2H, 2 × CH=C), 7.29 (s, 2H, 2 × CH=C), 7.32 (m, 2H, 2 × CH—N), 7.34 (m, 2H, 2 × CH—N), 7.36 (m, 2H, 2 × CH=C), 7.37 (s, 2H, 2 × CH=C), 7.40 (s, 2H, 2 × CH=C), 7.49 (d, 2H, 2 × CH=C, J=1.90 Hz), 8.08 (s, 2H, 2 × CH—N). IR (KBr) ν_max/_cm^−1^: 3123, 3100, 2977 (C—H), 1722 (C=N), 1588, 1563 (C—C, C=C), 1518 (C—C, C—H, C=C), 1471 (C—H, C=C), 1431 (C=C), 1380 (NO_3_), 1245, 1183, 1118, 1098 (C—O—C), 840, 831, 820 (C—Cl).

#### 3.2.2. Synthesis of [Ag(Miconazole)_2_ClO_4_] (**2**)

AgClO_4_ solution (0.5 mmol, 0.104 g) in 10 mL of ethanol was added to the miconazole ethanolic solution (miconazole (1 mmol, 0.416 g) in 10 mL of ethanol). Next, the whole mixture was stirred for 24 h at room temperature. The final product was filtered off and washed with portions of diethyl ether. The white crystals suitable for X-ray diffraction were grown from pure acetonitrile at room temperature. M_W_ 1038.92 g/mol; yield: (0.48 g, 92%); m.p. 175–177 °C. ESI-MS (methanol) *m*/*z*: 938.87 [Ag(MCZ)_2_]^+^. El. anal. Measured (calc.%): C—41.68 (41.62); H—2.36 (2.71); N—5.41 (5.40). ^1^H-NMR (600 MHz, CDCl_3_) δ (ppm): 4.16 (m, 4H, 2 × N—CH_2_), 4.19 (m, 2H, 2 × CH—O), 4.56 (s, 4H, 2 × CH_2_—O), 7.09 (s, 2H, 2 × CH=C), 7.29 (s, 2H, 2 × CH=C), 7.33 (m, 2H, 2 × CH—N), 7.34 (m, 2H, 2 × CH—N), 7.35 (m, 2H, 2 × CH=C), 7.39 (s, 2H, 2 × CH=C), 7.40 (s, 2H, 2 × CH=C), 7.49 (d, 2H, 2 × CH=C, J=1.90 Hz), 8.20 (s, 2H, 2 × CH—N). IR (KBr) ν_max/_cm^−1^: 3125, 3092, 2972 (C—H) 1727 (C=N), 1590, 1562 (C—C, C=C), 1526 (C—C, C—H, C=C), 1470 (C—H, C=C), 1437 (C=C), 1248, 1139, 1119, 1095 (C—O—C), 1078 (ClO_4_), 848, 816 (C—Cl).

### 3.3. Light Stability of Compounds ***1*** and ***2***

The light stability of the [Ag(MCZ)_2_NO_3_] and [Ag(MCZ)_2_ClO_4_] complexes, as well as silver(I) salts, was studied in normal daylight in air atmosphere and in the dark [20,55]. The complexes were dissolved in acetonitrile, while the silver(I) salts were dissolved in water. The concentration of each solution was 0.05 mol/L. For comparative purposes, the solutions of the complexes and silver(I) salts were applied onto various substrates (tissue paper, paper, glass, and synthetic leather) in the amount of 2.5 μmol (50 μL of the solution).

Stability was monitored visually for 108 h making image documentation after 0, 1, 4, 18, 24, 40, 48, 52, 60, 84, and 108 h. The photos of the samples are shown in the (Supplementary Materials Figure S3).

### 3.4. X-ray Single-Crystal Diffraction

The crystallographic data were collected using a SuperNova diffractometer with an Atlas detector using Cu*K*_α_ at 293 K for **1** and Mo*K*_α_ radiation at 100 K for **2**. Data reduction and multi-scan absorption correction were performed. The data for both complexes were collected and processed using CrysAlis PRO [58]. The structures were solved with SHELXT [59] and refined with SHELXL-2018/3 [60]. All non-hydrogen atoms were refined anisotropically. All (C)–H atoms of metronidazole ligands were calculated to their idealized positions and refined as riding atoms with isotropic displacement parameters *U*_iso_(H) = 1.2*U*_eq_(C). One oxygen atom (O2) in the nitrate group and one chlorine atom (Cl1) of structure **1** were found to be disordered and refined in two alternative positions with the final site occupation factors equal to 0.5. In structure **2**, one oxygen atom (O3) in the perchlorate group was also refined as disordered (0.5). Within the disordered parts, geometrical similarity restraints were applied to some N–O, C–Cl bonds in **1** and Cl–O bonds in **2** using the SADI instruction.

### 3.5. QTAIM

Single-point calculations of **1**–**2** and five Ag(I) complexes of metronidazole [20] with the X-ray single-crystal coordinates (the distances to H-atoms were normalized [61]) were carried out using Gaussian09 [62] at the B3PW91 level [63,64] using effective core potentials along with the associated VTZ basis set for Ag [65,66] and 6-311+G(d) basis elsewhere. Subsequently, the wave function files were used for a topological analysis of the electron density with AIMALL [67].

### 3.6. Chemistry

The ^1^H-NMR spectra were recorded with a Bruker Advance III 600 MHz spectrophotometer (Bruker Corporation, Billerica, MA, USA) in CDCl_3_ at room temperature. Infrared spectra were recorded in the range of 4000–400 cm^−1^ on a Bruker Alpha-T (Bruker Corporation, Billerica, MA, USA) using KBr pellets. Electrospray ionization mass spectra (ESI-MS) were registered in positive ion mode on a Varian 500-MS LC Trap (Varian Inc., Palo Alto, CA, USA). Elemental analyses (C, H, N) were performed at the Faculty of Pharmacy (Medical University of Lodz, Poland) with a Perkin Elmer 2400 analyzer (PerkinElmer, Waltham, MA, USA). Melting points of all the compounds were determined with a Böetius apparatus.

### 3.7. Cell Culture and Cytotoxicity Assessment

#### 3.7.1. Cell Culture

The human hepatoma cell line (HepG2) was purchased from the American Type Culture Collection (ATCC HB-8065). These cells were cultured in Minimum Essential Medium Eagle (MEME) (ATCC). The murine fibroblast cell line (Balb/c 3T3 clone A31) was purchased from the American Type Culture Collection (ATCC CCL-163), and the cells were cultured in Dulbecco’s Modified Eagle’s Medium (DMEM) (ATCC). The media were supplemented with 10% BCS (Balb/c 3T3), 10% FBS (HepG2), 1% l-glutamine, and 1% antibiotic solution. The cells were maintained in 75-cm^2^ cell culture flasks (NUNC, Roskilde, Denmark) in a humidified incubator, NuAire (Plymouth, MN, USA) at 37 °C and 5% CO_2_. The medium was refreshed every two days and the cells were trypsinized by 0.25% trypsin–0.02% EDTA after reaching 70–80% confluence. Then, cell suspensions at a density of 2 × 10^5^ cells/mL (HepG2) and 5 × 10^4^ cells/mL (Balb/c 3T3) were transferred to 96-well plates (100 μL/well) and incubated for 24 h before the exposure to the studied compounds.

#### 3.7.2. Compounds Preparation and Exposure

Stock solutions of ligands, cisplatin, and silver(I) miconazole complexes were dissolved in DMSO and diluted with the culture medium to obtain a concentration range from 10^−4^ to 10^0^ μM. The final concentration of DMSO in the medium was 0.1% and had no influence on cell growth. The medium used for test solutions and controls did not contain serum and antibiotics. As a control, cultured cells were grown in the absence of the studied compounds. Each concentration was tested in six replicates in three independent experiments. Cytotoxicity was assessed after 72 h of cell exposure to the compounds in darkness. The medium was not changed during the incubation time.

#### 3.7.3. Cytotoxicity Assessment

**MTT assay.** The metabolic activity of living cells was assessed by the measurement of the activity of dehydrogenases [38]. After incubation of the cells with the study compound, 10 μL of the MTT solution (5 mg/mL in phosphate buffered saline (PBS)) was added to each well of the 96-well plate and incubated. After 3 h, the MTT solution was removed, and intracellular formazan crystals were dissolved in 100 μL DMSO. The plate was shaken for 15 min at room temperature and transferred to a microplate reader (Synergy HTX multi-mode reader (BioTek^®^ Instruments Inc., Winooski, VT, USA)) to measure the absorbance at 570 nm, using a blank as a reference. Cytotoxicity was expressed as a percentage of the negative control (0.1% DMSO) [33].

**NRU assay**. This method is based on staining living cells with neutral red which readily diffuses through the plasma membrane and accumulates in lysosomes [36]. After the incubation, the medium containing the drug was removed, and the cells were washed with PBS. Then, 100 μL/well of NR solution (50 μg/mL) was added for 3 h. After this time, the cells were washed with PBS. The dye from viable cells was released by extraction with a mixture of acetic acid, ethanol, and water (1:50:49, *v*:*v*:*v*). After 10 min of shaking, the absorbance of the dissolved NR was measured using a Synergy HTX multi-mode reader (BioTek^®^ Instruments Inc., Winooski, VT, USA) at 540 nm using a blank as a reference. Cytotoxicity was expressed as a percentage of the negative control (0.1% DMSO) [33].

**TPC assay**. The assay was based upon staining total cellular protein (proliferation) [37]. After the incubation, the medium containing drug was removed and 100 μL of Coomassie brilliant blue R-250 dye was added to each well. The plate was shaken for 10 min. Then, the stain was removed and the cells were rinsed twice with 100 μL of washing solution (glacial acetic acid/ethanol/water, 5:10:85, *v*:*v*:*v*). After that, 100 μL of the desorbing solution (1 M potassium acetate) was added, and plates were shaken again for 10 min. The absorbance was measured at 595 nm in a microplate reader (Synergy HTX multi-mode reader (BioTek^®^ Instruments Inc., Winooski, VT, USA)) using a blank as a reference. Cytotoxicity was expressed as a percentage of the negative control (0.1% DMSO) [33].

**LDH leakage assay**. The integrity of the plasma membrane was assessed through the test of lactate dehydrogenase (LDH) release [39], which was monitored using the commercially available Cytotoxicity Detection Kit (LDH) (Roche Diagnostics, Warsaw, Poland). The medium (100 μL/well) without cells was transferred into the corresponding wells of an optically clear 96-well flat-bottom microplate, and 100 μL of reaction mixture was added to each well. Then, the plates were incubated for 30 min at room temperature in darkness. After that time, 50 μL/well of 1 M HCl was added to stop the reaction. The absorbance was measured at 492 nm in a microplate reader (Synergy HTX multi-mode reader (BioTek^®^ Instruments Inc., Winooski, VT, USA)) using a blank as a reference [33].

#### 3.7.4. Statistical Analysis

The study was performed in three independent experiments. The obtained results are presented as mean values ± SD (standard deviation). Assessment of the cytotoxicity data used a one-way analysis of variance (ANOVA) followed by Dunnett’s post-hoc test, which was applied to determine significance relative to the negative control. The cytotoxicity concentration (IC_20_ and IC_50_) necessary for 20% and 50% inhibition of cell viability, respectively, by the drug was calculated using GraphPad Prism 5.0. Statistical comparisons among IC_50_ values were performed by the analysis of variance (ANOVA) followed by the Tukey test, and differences were considered statistically significant at *p* ≤ 0.05.

## 4. Conclusions

This paper proposed a facile and convenient method for synthesis of silver(I) complexes using miconazole and silver(I) nitrate and silver(I) perchlorate. The developed direct method of synthesis can be considered as simple, fast, and accurate. The present work demonstrated the use of two medicines, i.e., miconazole and silver(I) nitrate, in the direct synthesis of the complexes for the evaluation of their cytotoxic activity.

The results of our research show that complexes of silver(I) ions with the biologically active ligand miconazole inhibit the growth of HepG2 cancer cells.

The tested compounds were much more toxic to human cancer cells compared to the free ligand. In addition, the obtained silver(I) complexes more powerfully eliminated HepG2 cancer cells than cisplatin. Miconazole and silver salts were not toxic to the cell lines tested. Moreover, hepatic cancer HepG2 cells were more sensitive to the action of silver(I) complexes than the fibroblasts. The results indicated better efficiency of elimination of HepG2 cancer cells and lower toxicity to Balb/c 3T3 fibroblasts for [Ag(MCZ)_2_NO_3_] compared to the silver(I) complex of metronidazole [Ag(MTZ)_2_NO_3_] [57].

Based on the chemical bonding analysis, it seems that the different molecular conformations of the analyzed complexes and the strength of interaction between the cation and anion did not affect the cytotoxic activity of the complex compounds, since they exhibited a similar effect on cancer cells, leading to the disintegration of cellular membranes. The new compounds could be probable candidates as promising therapeutic agents for the treatment of hepatocellular carcinoma, which should attract further studies. 

Miconazole silver(I) complexes may be considered as a therapeutic option in skin infections caused by fungal strains, as well as by bacteria strains that are resistant to antibiotics. After all, we recently reported [68] the significant effect of treatment of patients with ocular rosacea with a silver(I) complex of metronidazole, which might be an alternative method of acne rosacea treatment. 

Evaluation of the microbiological activity of silver(I) miconazole complexes will be the subject of our further study.

## Figures and Tables

**Table 1:** Proton chemical shifts in ppm for miconazole and complex compounds at room temperature in CDCl_3_.

	Miconazole	Ag(MCZ)_2_NO_3_	Ag(MCZ)_2_ClO_4_
Proton	Chemical Shift (ppm)	Chemical Shift (ppm)	Chemical Shift (ppm)
Imidazole			
H2	-	8.08	8.20
H4	7.26	7.32	7.33
H5	7.27	7.34	7.34
2,4-Dichlorophenyl			
(Ring I) H3	7.37	7.40	7.40
(Ring I) H5	7.20	7.29	7.29
(Ring I) H6	7.16	7.08	7.09
(Ring II) H3′	7.49	7.49	7.49
(Ring II) H5′	7.33	7.37	7.39
(Ring II) H6′	7.32	7.36	7.35
Aliphatic-CH	4.10	4.16	4.19
2-Aliphatic-CH_2_	4.27–4.52	4.29–4.55	4.32–4.56

**Table 2:** Crystallographic data for **1** and **2**.

	1	2
Empirical formula	C_36_H_28_AgCl_8_N_5_O_5_	C_36_H_28_AgCl_8_N_4_O_2_·ClO_4_
Formula weight	1002.10	1039.54
Crystal system	Monoclinic	Monoclinic
Space group	*C*2/c	*C*2/c
*a* (Å)	25.8806(15)	15.6640(6)
*b* (Å)	8.0278(5)	8.5524(3)
*c* (Å)	19.8754(15)	30.2426(12)
α (°)	90.000	90.000
β (°)	103.273(7)	97.335(4)
γ (°)	90.000	90.000
*V* (Å^3^)	4019.1(5)	4018.3(3)
*Z*	4	4
*T* (K)	293(1)	100(1)
*F* (000)	2008	2080
*D_x_* (g∙cm^−3^)	1.656	1.718
*µ* (mm^−1^)	9.33	1.15
Wavelength (Å)	1.54184	0.71073
*θ* range (°)	3.5–68.0	3.0–29.0
Measured reflections	8223	19996
Unique reflections	3561	5346
Observed reflections [*I* > 2*σ* (*I*)]	2035	4518
Completeness to *θ*_max_ (%)	97.2	99.8
Parameters/restraints	269/14	251/1
*R* [*F*^2^ > 2*σ* (*F*^2^)]	0.059	0.054
*wR* (all data)	0.181	0.143
*S*	1.06	1.06

**Table 3:** Geometry (Å, °) around silver(I) in complexes **1** and **2**.

	1	2		1	2
**Bond Lengths**			**Bond Angles**		
Ag–N	2.181(5)	2.068(3)	N–Ag–N^i^	152.4(3)	175.81(16)
Ag–N^i^	2.181(5)	2.068(3)	N–Ag–O_nitrate_	123.4(4)	-
Ag–O_nitrate_	2.510(15)	-	N^i^–Ag1–O_nitrate_	84.2(4)	-
Ag–O_chlorate_	-	3.140(3)	N–Ag–O_chlorate_	-	99.91(17)
Ag–Cl^ii^	3.189(2)	-	N^i^–Ag–O_chlorate_	-	84.02(17)

Symmetry codes: for **1** (i) 1 − *x*, *y*, ½ − *z*; (ii) 1 − *x*, −*y*, 1 − *z*; for **2** (i) 1 − *x*, *y*, ½ − *z*.

**Table 4:** Hydrogen bonding geometry (Å, °) for silver(I) complexes **1** and **2**.

H–Bond	D–H	H⋯A	D⋯A	D–H⋯A
**1**				
C3–H3⋯O3^iv^	0.93	2.36	3.202(8)	150
C17–H17⋯O2A^iv^	0.93	2.64	3.436(19)	144
C17–H17⋯O2B^iv^	0.93	2.53	3.363(19)	149
C18–H18⋯O2A^v^	0.93	2.67	3.347(19)	130
C18–H18⋯O2B^v^	0.93	2.60	3.415(19)	146
C15–H15⋯*Cg*(1)^ii^	0.93	2.94	3.798(8)	155
**2**				
C2–H2A⋯O2^ii^	0.99	2.67	3.531(5)	145
C2–H2A⋯O3A^iii^	0.99	2.70	3.474(7)	135
C2–H2A⋯O3B^ii^	0.99	2.52	3.471(7)	161
C2–H2A⋯O3B^iii^	0.99	2.54	3.388(7)	144
C3–H3⋯O2^i^	0.95	2.51	3.356(5)	136
C4–H4⋯O3A^iv^	0.95	2.65	3.579(7)	166
C4–H4⋯O3B^iv^	0.95	2.55	3.372(7)	145

In **1**: *Cg*(1)—center of gravity of the heterocyclic ring. Symmetry codes: **1** (ii) 1 − *x*, − *y*, 1 − *z*; (iv) *x*, − 1 + *y*, *z*; (v) 1 − *x*, − 1 + *y*, ½ − *z*; **2** (i) 1 − *x*, *y*, ½ − *z* (ii) − ½ + *x*, − ½ + *y*, *z*; (iii) ½ − *x*, − ½ + *y*, ½ − *z*; (iv) *x*, − 1 + *y*, *z.*

**Table 5:** Quantum Theory of Atoms in Molecules (QTAIM) bonding indicators for Ag−N and Ag−O/F for silver(I) complexes with metronidazole and miconazole ligands and different counter ions.

	*d*	N–Ag–N/O	*ρ*_bcp_	∇^2^*ρ*_bcp_	*ε*	*G/ρ*_bcp_	*H*/*ρ*_bcp_	*δ*
**Ag**–**N**								
(**2**) MCZ-ClO_4_^−^	2.068	176	0.68	9.3	0.05	1.23	−0.28	0.66
MTZ-BF_4_^−^	2.110	175	0.62	8.5	0.05	1.21	−0.25	0.62
MTZ-ClO_4_^−^	2.126	172	0.60	8.2	0.05	1.20	−0.24	0.59
MTZ-NO_3_^−^	2.147	165	0.57	7.9	0.04	1.19	−0.22	0.59
MTZ-CF_3_COO^−^	2.160	163	0.55	7.7	0.03	1.19	−0.21	0.57
MTZ-CH_3_SO_3_^−^	2.165	170	0.55	7.6	0.04	1.19	−0.21	0.56
(**1**) MCZ-NO_3_^−^	2.181	152	0.53	7.5	0.02	1.20	−0.20	0.55
**Ag**–**O**	*d*	N−Ag−O_anion_	*ρ*_bcp_	∇^2^*ρ*_bcp_	*ε*	*G/ρ*_bcp_	*H*/*ρ*_bcp_	*δ*
MTZ-CF_3_COO^−^	2.480	112	0.25	3.6	0.04	1.03	−0.01	0.29
(**1**) MCZ-NO_3_^−^	2.510	123	0.22	3.6	0.04	1.12	0.00	0.17
MTZ-CH_3_SO_3_^−^	2.577	100	0.20	2.8	0.07	0.97	−0.01	0.23
MTZ-ClO_4_^−^	2.660	91	0.17	2.3	0.07	0.93	0.01	0.18
MTZ-NO_3_^−^	2.669	112	0.17	2.2	0.07	0.91	0.01	0.19
(**2**) MCZ-ClO_4_^−^	3.140	100	0.07	0.8	0.07	0.75	0.03	0.07
**Ag**–**F**	*d*	N−Ag−F_anion_	*ρ*_bcp_	∇^2^*ρ*_bcp_	*ε*	*G/ρ*_bcp_	*H*/*ρ*_bcp_	*δ*
MTZ-BF_4_^−^	2.885		0.09	1.2	0.03	0.92	0.04	0.09

**Table 6:** Cytotoxicity concentrations (IC_20_ and IC_50_, μM) determined in HepG2 Balb/c 3T3 cells by the 3-(4,5-dimethyl-2-thiazolyl)-2,5-diphenyl-2-H-tetrazolium bromide (MTT), neutral red uptake (NRU), total protein content (TPC), and lactate dehydrogenase (LDH) assays after 72 h of exposure to the studied compounds; data are presented as means (*n* = 3) ± SD.

	HepG2				Balb/c 3T3			
	MTT	NRU	TPC	LDH	MTT	NRU	TPC	LDH
				IC_20_			
**[Ag(MCZ)_2_NO_3_] **	0.14 ^a^ ± 0.02	0.16 ^a^ ± 0.01	0.09 ^a^ ± 0.04	0.08 ^a^ ± 0.01	0.25 ^b^ ± 0.03	0.23 ^b^ ± 0.02	0.22 ^b^ ± 0.04	0.18 ^b^ ± 0.01
**[Ag(MCZ)_2_ClO_4_]**	0.12 ^a^ ± 0.02	0.14 ^a^ ± 0.02	0.10 ^a^ ± 0.01	0.06 ^a^ ± 0.01	0.24 ^b^ ± 0.01	0.26 ^b^ ± 0.03	0.21 ^b^ ± 0.01	0.22 ^b^ ± 0.03
**Miconazole**	> 1	> 1	> 1	> 1	> 1	> 1	> 1	> 1
**AgNO_3_**	> 1	> 1	> 1	> 1	> 1	> 1	> 1	> 1
**AgClO_4_**	> 1	> 1	> 1	> 1	> 1	> 1	> 1	> 1
**Cisplatin**	< 0.0001	< 0.0001	< 0.0001	0.01 ± 0.001	> 1	> 1	> 1	> 1
					IC_50_			
**[Ag(MCZ)_2_NO_3_]**	0.46 ^a^ ± 0.01	0.47 ^a^ ± 0.02	0.45 ^a^ ± 0.02	0.40 ^a^ ± 0.01	0.54 ^b^ ± 0.02	0.54 ^b^ ± 0.01	0.53 ^b^ ± 0.01	0.52 ^b^ ± 0.04
**[Ag(MCZ)_2_ClO_4_]**	0.45 ^a^ ± 0.01	0.46 ^a^ ± 0.01	0.44 ^a^ ± 0.02	0.26 ^a^ ± 0.08	0.53 ^b^ ± 0.01	0.53 ^b^ ± 0.02	0.52 ^b^ ± 0.02	0.51 ^b^ ± 0.03
**Miconazole**	> 1	> 1	> 1	> 1	> 1	> 1	> 1	> 1
**AgNO_3_**	> 1	> 1	> 1	> 1	> 1	> 1	> 1	> 1
**AgClO_4_**	> 1	> 1	> 1	> 1	> 1	> 1	> 1	> 1
**Cisplatin**	> 1	> 1	> 1	0.01 ± 0.001	> 1	> 1	> 1	> 1

The different letters (a, b) within lines indicate significant differences (*p* ≤ 0.05) between the cell cultures for the corresponding assay; <0.0001—the IC_20_ value was under 0.0001 μM (the lowest concentration tested); >1—the IC_20_ or IC_50_ value was over 1 μM (the highest concentration tested).

## Supplementary Materials

Supplementary materials can be found at https://www.mdpi.com/1422-0067/21/10/3629/s1.
