# Supplementary material for: Synthesis, Spectroscopy, Light Stability, Single-Crystal Analysis, and In Vitro Cytotoxic Activity on HepG2 Liver Cancer of Two Novel Silver(I) Complexes of Miconazole
Source: Int J Mol Sci. 2020 May 21;21(10):3629. doi: 10.3390/ijms21103629 (PMC7279231; doi:10.3390/ijms21103629)

checkCIF/PLATON report

Structure factors have been supplied for datablock(s) complex1, complex2

THIS REPORT IS FOR GUIDANCE ONLY. IF USED AS PART OF A REVIEW PROCEDURE FOR PUBLICATION, IT SHOULD NOT REPLACE THE EXPERTISE OF AN EXPERIENCED CRYSTALLOGRAPHIC REFEREE.

No syntax errors found. CIF dictionary Interpreting this report

Datablock: complex1

```
Bond precision:  C-C = 0.0094 A          Wavelength=1.54184

Cell:           a=25.8806(15)    b=8.0278(5)        c=19.8754(15)
                alpha=90         beta=103.273(7)    gamma=90
Temperature:    293 K

                Calculated                 Reported
Volume          4019.1(5)                  4019.1(5)
Space group     C 2/c                      C 2/c
Hall group      -C 2yc                     -C 2yc
Moiety formula  C36 H28 Ag Cl8 N5 O5       C36 H28 Ag Cl8 N5 O5
Sum formula     C36 H28 Ag Cl8 N5 O5       C36 H28 Ag Cl8 N5 O5
Mr              1002.10                    1002.10
Dx,g cm-3       1.656                      1.656
Z               4                          4
Mu (mm-1)       9.331                      9.331
F000            2008.0                     2008.0
F000'           2024.59
h,k,lmax        30,9,23                    30,9,23
Nref            3663                       3561
Tmin,Tmax       0.375,0.393                0.626,1.000
Tmin'           0.053

Correction method= # Reported T Limits: Tmin=0.626 Tmax=1.000
AbsCorr = MULTI-SCAN

Data completeness= 0.972          Theta(max)= 67.999

R(reflections)= 0.0585( 2035)     wR2(reflections)= 0.1808( 3561)

S = 1.060                  Npar= 269
```

The following ALERTS were generated. Each ALERT has the format
test-name_ALERT_alert-type_alert-level.
Click on the hyperlinks for more details of the test.

Alert level C

```
PLAT029_ALERT_3_C _diffrn_measured_fraction_theta_full value Low .      0.973 Why?
PLAT234_ALERT_4_C Large Hirshfeld Difference O2A      --N3       .       0.16 Ang.
PLAT234_ALERT_4_C Large Hirshfeld Difference O2B      --N3       .       0.21 Ang.
PLAT234_ALERT_4_C Large Hirshfeld Difference O3       --N3       .       0.19 Ang.
PLAT242_ALERT_2_C Low    MainMol Ueq as Compared to Neighbors of         N3 Check
PLAT342_ALERT_3_C Low Bond Precision on  C-C Bonds ...............    0.00944 Ang.
PLAT906_ALERT_3_C Large K Value in the Analysis of Variance ......      4.050 Check
PLAT911_ALERT_3_C Missing FCF Refl Between Thmin & STh/L=    0.600        100 Report
```

Alert level G

```
PLAT002_ALERT_2_G Number of Distance or Angle Restraints on AtSite          6 Note
PLAT003_ALERT_2_G Number of Uiso or Uij Restrained non-H Atoms ...          2 Report
PLAT176_ALERT_4_G The CIF-Embedded .res File Contains SADI Records          2 Report
PLAT186_ALERT_4_G The CIF-Embedded .res File Contains ISOR Records          1 Report
PLAT199_ALERT_1_G Reported _cell_measurement_temperature ..... (K)        293 Check
PLAT200_ALERT_1_G Reported   _diffrn_ambient_temperature ..... (K)        293 Check
PLAT300_ALERT_4_G Atom Site Occupancy of Cl1A       Constrained at        0.5 Check
PLAT300_ALERT_4_G Atom Site Occupancy of Cl1B       Constrained at        0.5 Check
PLAT300_ALERT_4_G Atom Site Occupancy of O2A        Constrained at        0.5 Check
PLAT300_ALERT_4_G Atom Site Occupancy of O2B        Constrained at        0.5 Check
PLAT301_ALERT_3_G Main Residue  Disorder ..............(Resd  1  )         7% Note
PLAT793_ALERT_4_G Model has Chirality at C1          (Centro SPGR)          R Verify
PLAT860_ALERT_3_G Number of Least-Squares Restraints .............         14 Note
PLAT912_ALERT_4_G Missing # of FCF Reflections Above STh/L=  0.600          3 Note
PLAT978_ALERT_2_G Number C-C Bonds with Positive Residual Density.          1 Info
```

0 **ALERT level A** = Most likely a serious problem - resolve or explain
0 **ALERT level B** = A potentially serious problem, consider carefully
8 **ALERT level C** = Check. Ensure it is not caused by an omission or oversight
15 **ALERT level G** = General information/check it is not something unexpected

2 ALERT type 1 CIF construction/syntax error, inconsistent or missing data
4 ALERT type 2 Indicator that the structure model may be wrong or deficient
6 ALERT type 3 Indicator that the structure quality may be low
11 ALERT type 4 Improvement, methodology, query or suggestion
0 ALERT type 5 Informative message, check

Datablock: complex2

Bond precision: C-C = 0.0053 A   Wavelength=0.71073

Cell:	a=15.6640(6)	b=8.5524(3)	c=30.2426(12)
	alpha=90	beta=97.335(4)	gamma=90
Temperature:	100 K		

	Calculated	Reported
Volume	4018.3(3)	4018.3(3)
Space group	C 2/c	C 2/c
Hall group	-C 2yc	-C 2yc
Moiety formula	C36 H28 Ag Cl8 N4 O2, Cl O4	C36 H28 Ag Cl8 N4 O2, Cl O4
Sum formula	C36 H28 Ag Cl9 N4 O6	C36 H28 Ag Cl9 N4 O6
Mr	1039.54	1039.54
Dx,g cm-3	1.718	1.718
Z	4	4
Mu (mm-1)	1.151	1.151
F000	2080.0	2080.0
F000’	2082.40	
h,k,lmax	21,11,41	21,11,41
Nref	5355	5346
Tmin,Tmax	0.836,0.923	0.678,1.000
Tmin’	0.776	

Correction method= # Reported T Limits: Tmin=0.678 Tmax=1.000
AbsCorr = MULTI-SCAN

Data completeness= 0.998 Theta(max)= 28.999

R(reflections)= 0.0543(4518) wR2(reflections)= 0.1426(5346)

S = 1.058 Npar= 251

The following ALERTS were generated. Each ALERT has the format
test-name_ALERT_alert-type_alert-level.
Click on the hyperlinks for more details of the test.

Alert level C

```
PLAT906_ALERT_3_C Large K Value in the Analysis of Variance ......      3.193 Check
PLAT910_ALERT_3_C Missing # of FCF Reflection(s) Below Theta(Min).         7 Note
PLAT911_ALERT_3_C Missing FCF Refl Between Thmin & STh/L=    0.600         3 Report
PLAT971_ALERT_2_C Check Calcd Resid. Dens.  1.68A   From C18            2.33 eA-3
PLAT972_ALERT_2_C Check Calcd Resid. Dens.  0.45A   From O3B           -1.67 eA-3
```

Alert level G

```
PLAT002_ALERT_2_G Number of Distance or Angle Restraints on AtSite         3 Note
PLAT083_ALERT_2_G SHELXL Second Parameter in WGHT  Unusually Large     20.16 Why ?
PLAT171_ALERT_4_G The CIF-Embedded .res File Contains EADP Records         2 Report
PLAT176_ALERT_4_G The CIF-Embedded .res File Contains SADI Records         1 Report
PLAT231_ALERT_4_G Hirshfeld Test (Solvent)   Cl5       --O3A      .       19.6 s.u.
PLAT300_ALERT_4_G Atom Site Occupancy of O3A        Constrained at         0.5 Check
PLAT300_ALERT_4_G Atom Site Occupancy of O3B        Constrained at         0.5 Check
PLAT302_ALERT_4_G Anion/Solvent/Minor-Residue Disorder (Resd  2  )        40% Note
PLAT434_ALERT_2_G Short Inter HL..HL Contact Cl3       ..Cl3              3.20 Ang.
                                                 -x,1-y,1-z  =      5_566 Check
PLAT793_ALERT_4_G Model has Chirality at C1          (Centro SPGR)          R Verify
PLAT860_ALERT_3_G Number of Least-Squares Restraints .............         1 Note
```

```
PLAT933_ALERT_2_G Number of OMIT Records in Embedded .res File ...          3 Note
PLAT978_ALERT_2_G Number C-C Bonds with Positive Residual Density.          2 Info
```

```
  0 ALERT level A = Most likely a serious problem - resolve or explain
  0 ALERT level B = A potentially serious problem, consider carefully
  5 ALERT level C = Check. Ensure it is not caused by an omission or oversight
 13 ALERT level G = General information/check it is not something unexpected

  0 ALERT type 1 CIF construction/syntax error, inconsistent or missing data
  7 ALERT type 2 Indicator that the structure model may be wrong or deficient
  4 ALERT type 3 Indicator that the structure quality may be low
  7 ALERT type 4 Improvement, methodology, query or suggestion
  0 ALERT type 5 Informative message, check
```

It is advisable to attempt to resolve as many as possible of the alerts in all categories. Often the minor alerts point to easily fixed oversights, errors and omissions in your CIF or refinement strategy, so attention to these fine details can be worthwhile. In order to resolve some of the more serious problems it may be necessary to carry out additional measurements or structure refinements. However, the purpose of your study may justify the reported deviations and the more serious of these should normally be commented upon in the discussion or experimental section of a paper or in the "special_details" fields of the CIF. checkCIF was carefully designed to identify outliers and unusual parameters, but every test has its limitations and alerts that are not important in a particular case may appear. Conversely, the absence of alerts does not guarantee there are no aspects of the results needing attention. It is up to the individual to critically assess their own results and, if necessary, seek expert advice.

Publication of your CIF in IUCr journals

A basic structural check has been run on your CIF. These basic checks will be run on all CIFs submitted for publication in IUCr journals (*Acta Crystallographica*, *Journal of Applied Crystallography*, *Journal of Synchrotron Radiation*); however, if you intend to submit to *Acta Crystallographica Section C* or *E* or *IUCrData*, you should make sure that full publication checks are run on the final version of your CIF prior to submission.

Publication of your CIF in other journals

Please refer to the *Notes for Authors* of the relevant journal for any special instructions relating to CIF submission.

PLATON version of 22/12/2019; check.def file version of 13/12/2019

Datablock complex1 - ellipsoid plot

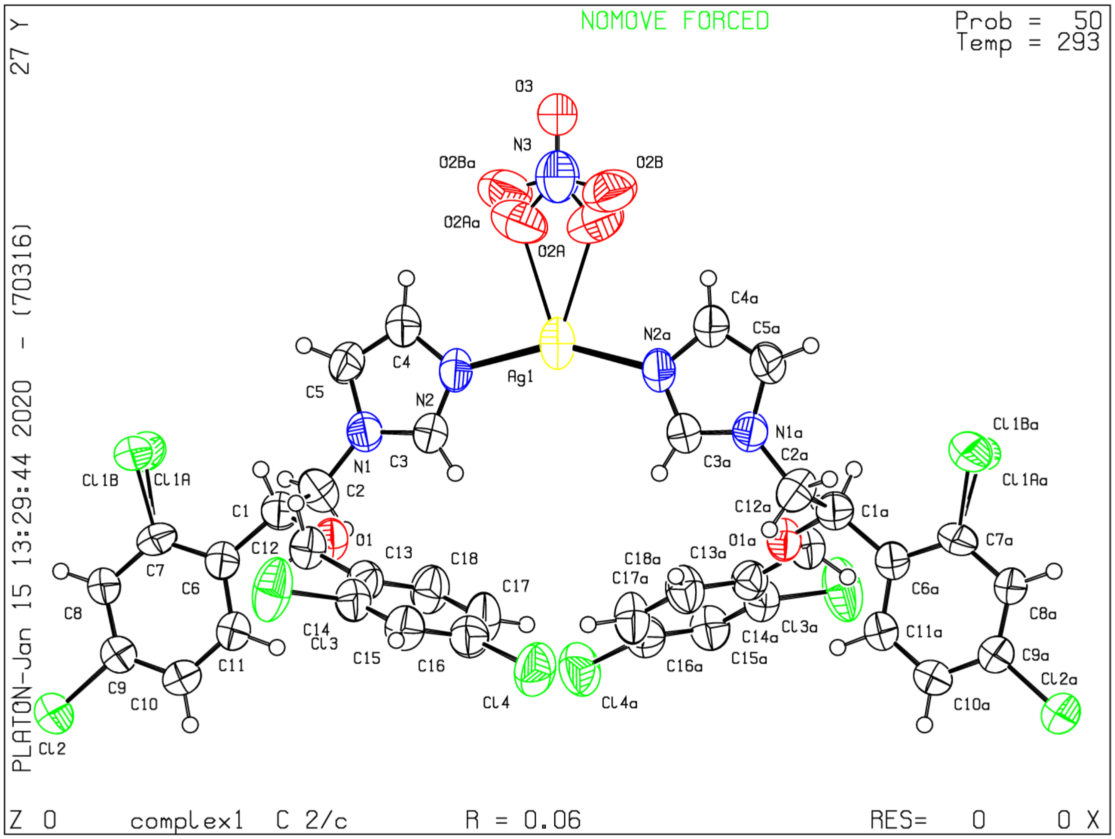

Datablock complex2 - ellipsoid plot

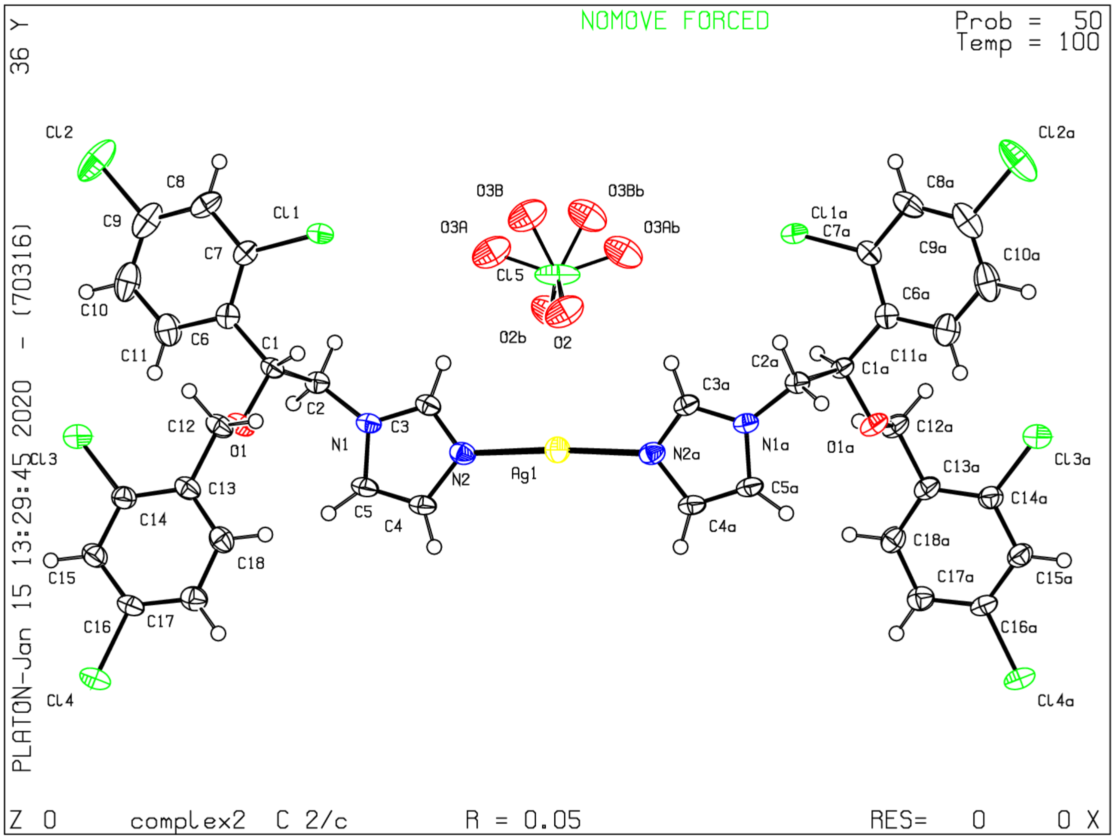

Supplement: Supplementary file 1 [file ijms-21-03629-s001.zip › ijms-797936 supplementary/supplementary files/checkcif-Ag-complexes-miconazole.pdf]
