# Supplementary material for: Synthesis, Spectroscopy, Light Stability, Single-Crystal Analysis, and In Vitro Cytotoxic Activity on HepG2 Liver Cancer of Two Novel Silver(I) Complexes of Miconazole
Source: Int J Mol Sci. 2020 May 21;21(10):3629. doi: 10.3390/ijms21103629 (PMC7279231; doi:10.3390/ijms21103629)
Supplement: Supplementary file 1 [file ijms-21-03629-s001.zip › ijms-797936 supplementary/supplementary files/revised Supplementary Materials ijms-797936.pdf]

Synthesis, spectroscopy, light stability, single crystal analysis and *in vitro* cytotoxic activity on HepG2 liver cancer of two novel silver(I) complexes of miconazole

Karolina Stryjska, Lidia Radko, Lilianna Chęcińska, Joachim Kusz, Andrzej Posyniak and Justyn Ochocki

Contents:

- **Figure S1.** 1H NMR spectra (600 MHz, $CDCl_3$) of miconazole (**a**), $[Ag(MCZ)_2NO_3]$ (**b**), $[Ag(MCZ)_2ClO_4]$ (**c**).
- **Figure S2.** IR spectra of miconazole (**a**), $[Ag(MCZ)_2NO_3]$ (**b**), $[Ag(MCZ)_2ClO_4]$ (**c**).
- **Figure S3.** Different substrates (tissue paper, paper, glass, synthetic leather) impregnated with 0.05 mol/l solutions of $AgNO_3$, $AgClO_4$, $[Ag(MCZ)_2NO_3]$, $[Ag(MCZ)_2ClO_4]$, exposed to in direct light and dark at room temperature.
- **Figure S4.** Molecular layers *ab* in the crystal structure of $[Ag(MCZ)_2ClO_4]$ (**2**).
- **Figure S5.** Concentration- and cell model-dependent decrease in viability after 72 h exposure to study compounds (miconazole, $AgNO_3$, $AgClO_4$ and cisplatin) assessed by MTT, NRU, TPC and LDH assays. The results are expressed as mean ±SD of three independent experiments. *p ≤0.05 in comparison with control.
- **Table S1.** Aromatic π…π interactions (Å,°) for silver(I) complexes 1 and 2.
- Final Cartesian coordinates (X, Y, Z in Å) for the gas-phase structure of the miconazole Ag(I) complex with NO_3 (**1**).
- Final Cartesian coordinates (X, Y, Z in Å) for the gas-phase structure of the miconazole Ag(I) complex with ClO_4 (**2**).
- Final Cartesian coordinates (X, Y, Z in Å) for the gas-phase structure of the metronidazole Ag(I) complex with BF_4-.
- Final Cartesian coordinates (X, Y, Z in Å) for the gas-phase structure of the metronidazole Ag(I) complex with CF_3COO^-.
- Final Cartesian coordinates (X, Y, Z in Å) for the gas-phase structure of the metronidazole Ag(I) complex with $CH_3SO_3^-$.
- Final Cartesian coordinates (X, Y, Z in Å) for the gas-phase structure of the metronidazole Ag(I) complex with ClO_4^-.
- Final Cartesian coordinates (X, Y, Z in Å) for the gas-phase structure of the metronidazole Ag(I) complex with NO_3^-.

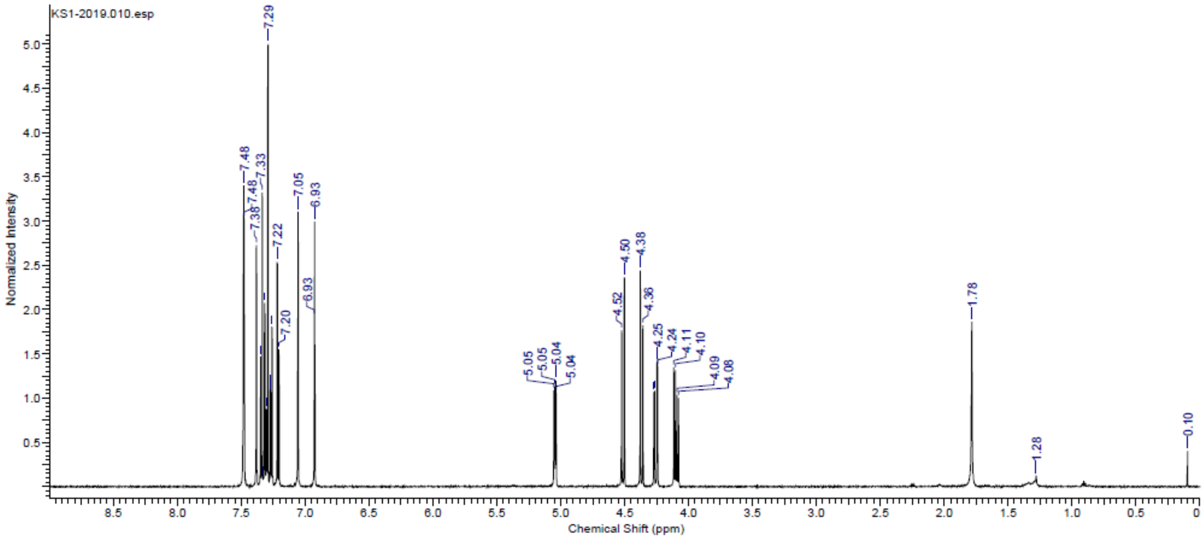

No.	(ppm)	(Hz)	Height	No.	(ppm)	(Hz)	Height	No.	(ppm)	(Hz)	Height	No.	(ppm)	(Hz)	Height	No.	(ppm)	(Hz)	Height
1	0.10	58.1	0.4007	10	4.27	2560.7	1.0808	19	5.05	3032.8	1.0872	28	7.27	4365.8	1.1503	37	7.38	4429.1	2.7270
2	1.28	768.5	0.1182	11	4.27	2563.4	1.0673	20	6.93	4157.3	2.9896	29	7.29	4376.4	5.0000	38	7.38	4431.3	2.6959
3	1.78	1071.1	1.8607	12	4.36	2615.0	1.8211	21	6.93	4158.4	1.9196	30	7.30	4381.6	0.8576	39	7.48	4487.4	3.0263
4	4.08	2447.8	1.0000	13	4.38	2627.8	2.4370	22	7.05	4234.8	3.1061	31	7.30	4383.5	0.8793	40	7.48	4489.7	3.3984
5	4.09	2455.3	1.0349	14	4.50	2702.7	2.3633	23	7.20	4324.4	1.5479	32	7.31	4389.9	1.9547	41	7.48	4491.2	3.0817
6	4.10	2462.5	1.3126	15	4.52	2715.5	1.7647	24	7.22	4332.7	2.5349	33	7.32	4391.8	2.0716				
7	4.11	2470.0	1.3460	16	5.04	3022.7	1.0541	25	7.26	4355.7	1.7937	34	7.33	4397.1	0.1077				
8	4.24	2546.1	1.3542	17	5.04	3025.3	1.1984	26	7.26	4357.5	1.8076	35	7.33	4402.7	3.3216				
9	4.25	2548.7	1.4103	18	5.05	3030.2	1.1333	27	7.27	4363.9	1.0690	36	7.35	4411.0	1.4680				

(a)

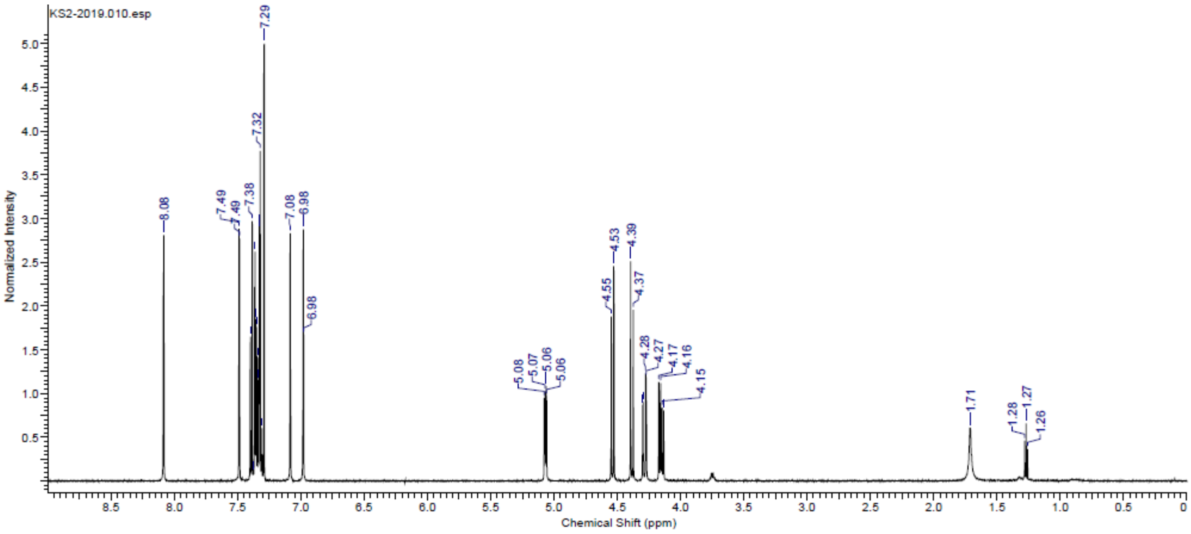

No.	(ppm)	(Hz)	Height	No.	(ppm)	(Hz)	Height	No.	(ppm)	(Hz)	Height	No.	(ppm)	(Hz)	Height	No.	(ppm)	(Hz)	Height
1	1.26	754.9	0.3643	10	4.28	2566.4	1.2292	19	5.07	3044.1	1.0240	28	7.32	4396.3	2.8809	37	7.38	4432.8	2.9692
2	1.27	762.1	0.6582	11	4.29	2578.1	0.9033	20	5.08	3046.7	0.9484	29	7.33	4398.2	2.9300	38	7.40	4441.1	1.6466
3	1.28	768.8	0.4592	12	4.30	2580.7	0.8674	21	6.98	4188.9	1.6741	30	7.34	4404.2	1.1504	39	7.49	4493.1	2.7699
4	1.71	1027.1	0.6097	13	4.37	2625.1	1.9584	22	6.98	4190.4	2.8744	31	7.34	4406.5	1.4049	40	7.49	4495.0	2.8811
5	4.13	2480.9	0.8075	14	4.39	2637.5	2.5102	23	6.98	4191.5	1.8601	32	7.35	4412.5	1.7598	41	8.08	4852.6	2.8087
6	4.15	2488.8	0.8384	15	4.53	2717.0	2.4526	24	7.08	4251.8	2.8293	33	7.35	4414.4	1.8460				
7	4.16	2495.6	1.1159	16	4.55	2729.4	1.8802	25	7.29	4376.4	5.0000	34	7.36	4418.9	2.6201				
8	4.17	2503.5	1.1308	17	5.06	3036.2	0.9684	26	7.31	4386.2	0.6013	35	7.36	4420.8	2.2193				
9	4.27	2563.4	1.1831	18	5.06	3038.8	1.0837	27	7.32	4394.4	3.7698	36	7.37	4426.8	0.1020				

(b)

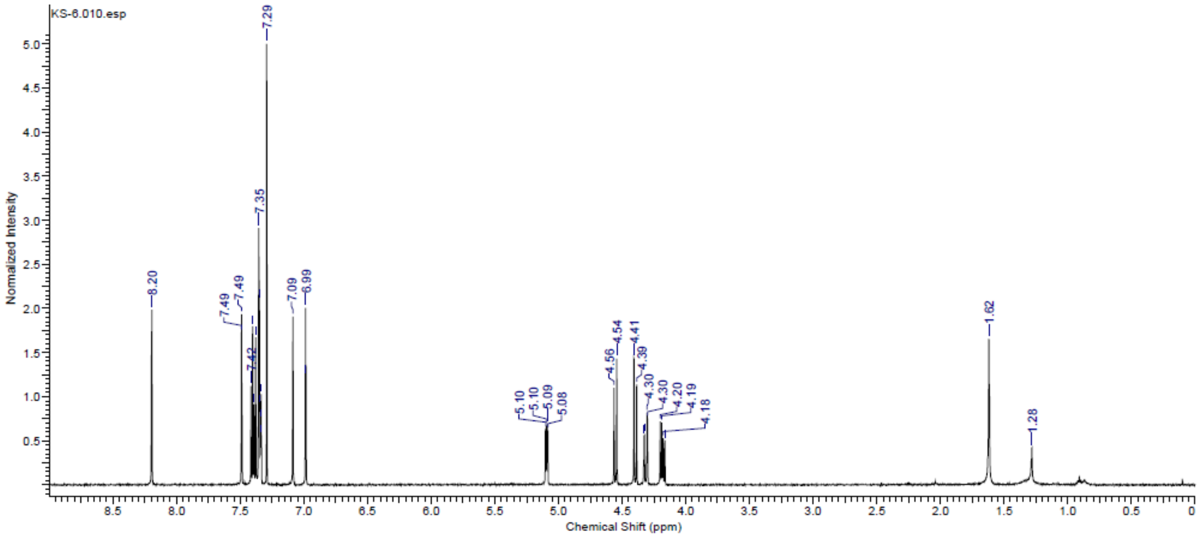

No.	(ppm)	(Hz)	Height	No.	(ppm)	(Hz)	Height	No.	(ppm)	(Hz)	Height	No.	(ppm)	(Hz)	Height	No.	(ppm)	(Hz)	Height
1	1.28	770.0	0.4189	9	4.32	2595.8	0.5743	17	5.10	3059.2	0.6740	25	7.34	4403.5	1.0262	33	7.40	4442.6	1.7978
2	1.62	971.8	1.6541	10	4.33	2598.4	0.5566	18	5.10	3061.8	0.6320	26	7.34	4404.2	0.9632	34	7.42	4451.3	1.1186
3	4.16	2499.4	0.5051	11	4.39	2632.6	1.1373	19	6.98	4191.9	1.3409	27	7.34	4406.5	0.7406	35	7.49	4494.2	1.9315
4	4.18	2507.3	0.5303	12	4.41	2645.4	1.4291	20	6.99	4193.4	2.0048	28	7.35	4409.5	0.9036	36	7.49	4496.1	1.7297
5	4.19	2514.1	0.7049	13	4.54	2726.4	1.4305	21	6.99	4194.5	1.2386	29	7.35	4411.8	2.0967	37	8.20	4919.2	1.9818
6	4.20	2522.0	0.7181	14	4.56	2738.8	1.0975	22	7.09	4253.3	1.9091	30	7.35	4414.0	2.9093				
7	4.30	2581.5	0.7505	15	5.08	3051.3	0.6169	23	7.29	4376.7	5.0000	31	7.38	4428.3	1.6715				
8	4.30	2584.1	0.8146	16	5.09	3054.3	0.6936	24	7.33	4401.6	0.5628	32	7.39	4436.6	0.9123				

(c)

Figure S1. ^1H NMR spectra (600 MHz, $CDCl_3$) of miconazole **(a)**, $[Ag(MCZ)_2NO_3]$ **(b)**, $[Ag(MCZ)_2ClO_4]$ **(c)**.

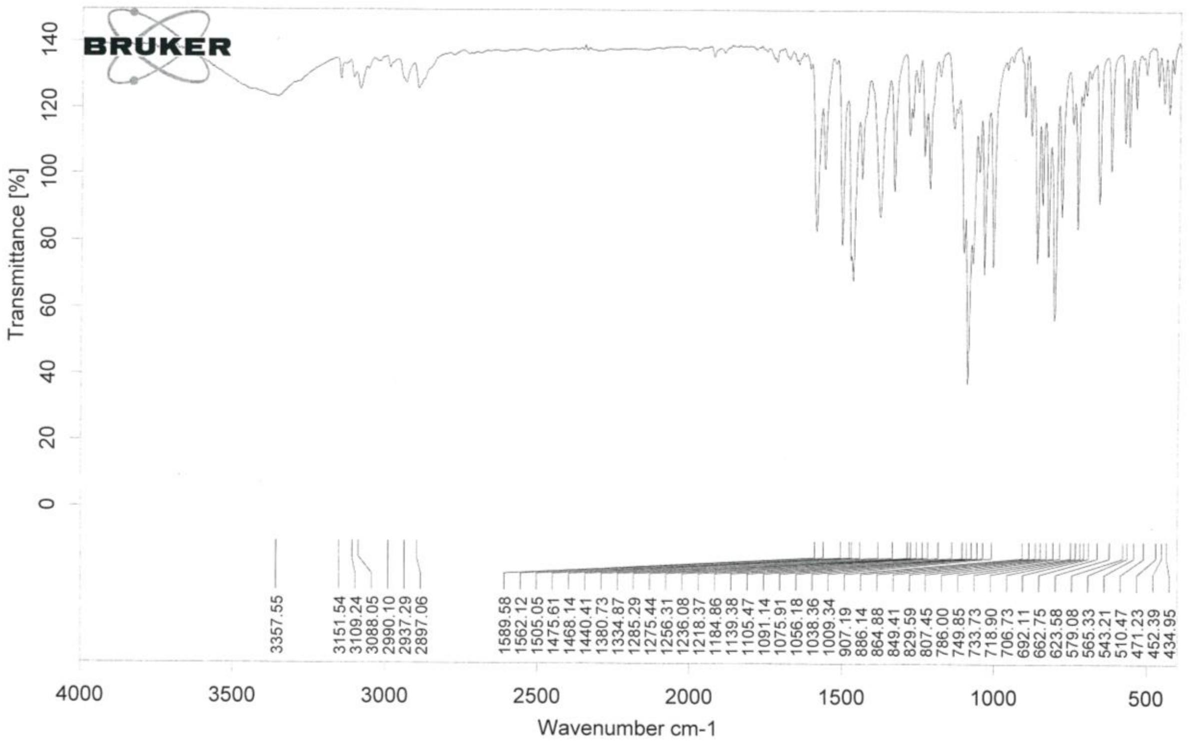

(a)

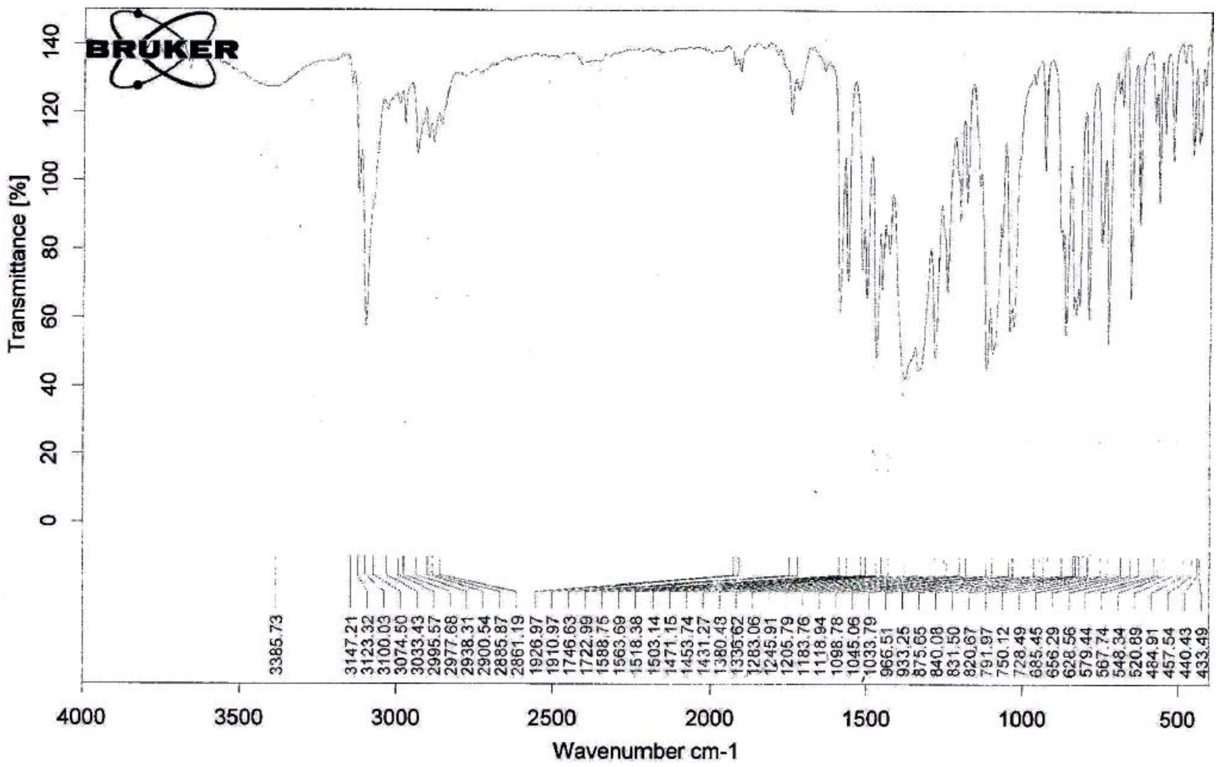

(b)

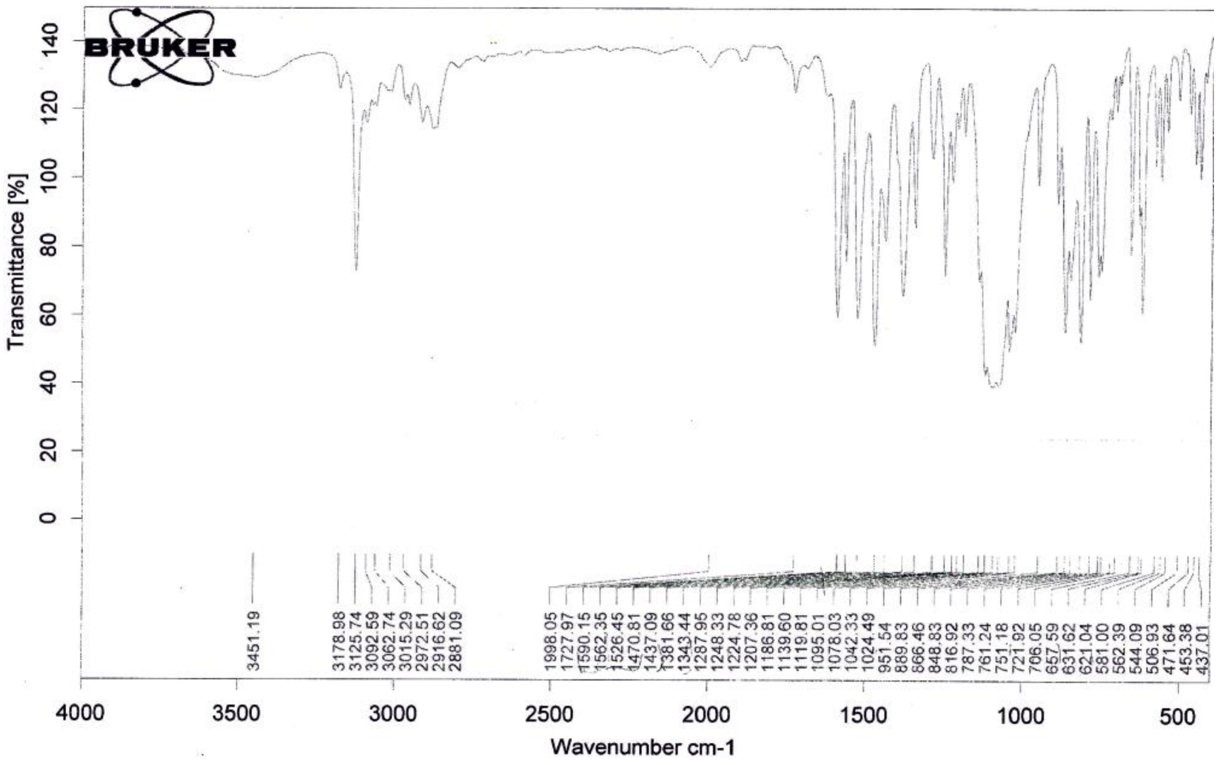

(c)

Figure S2. IR spectra of miconazole **(a)**, $[Ag(MCZ)_2NO_3]$ **(b)**, $[Ag(MCZ)_2ClO_4]$ **(c)**.

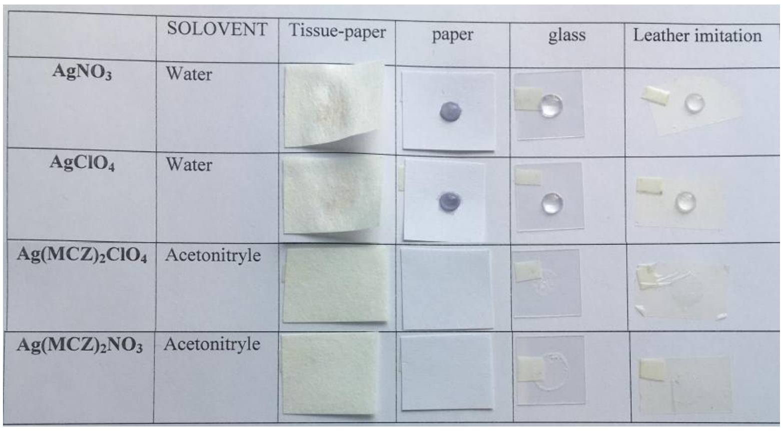

exposition time 0h –light

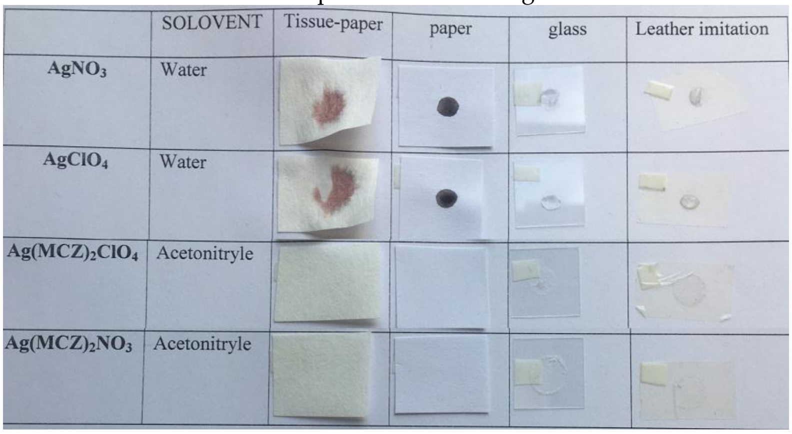

exposition time **1h –light**

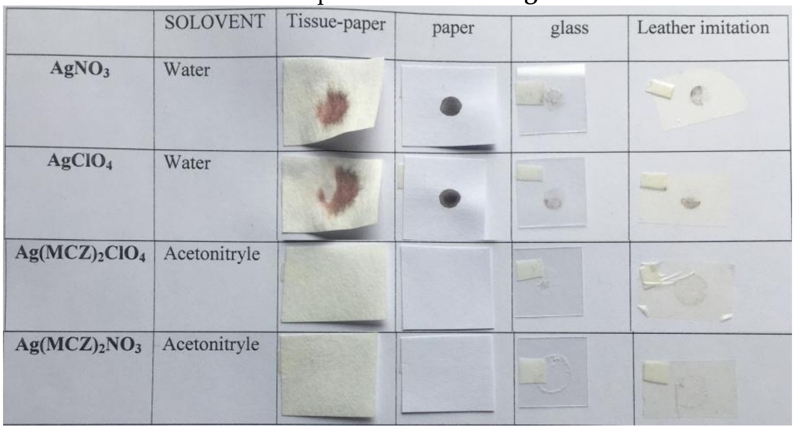

exposition time **4h –light**

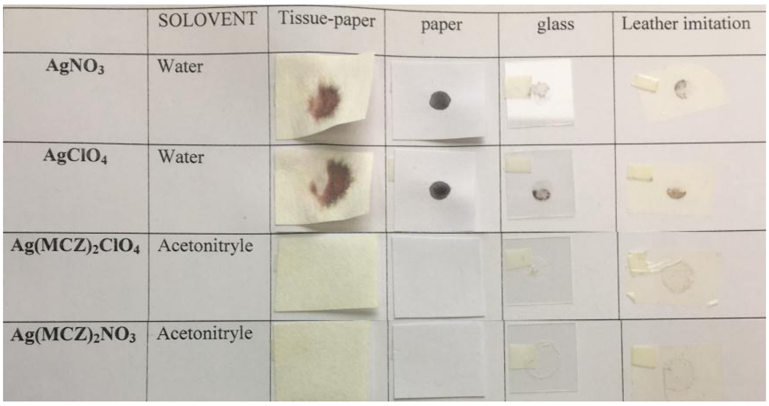

exposition time **18h –light**

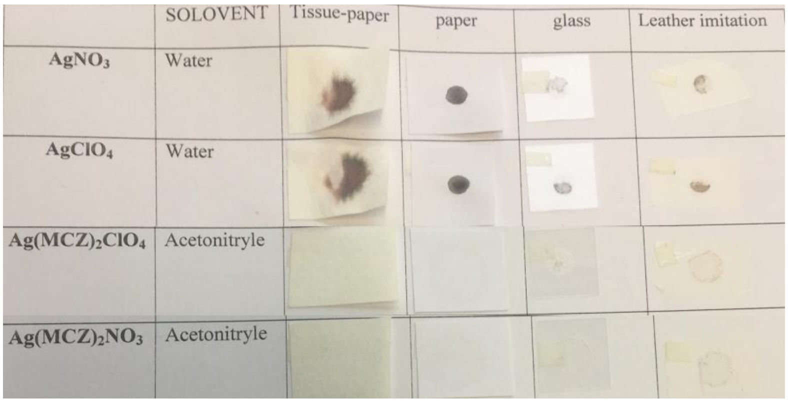

exposition time **24h –light**

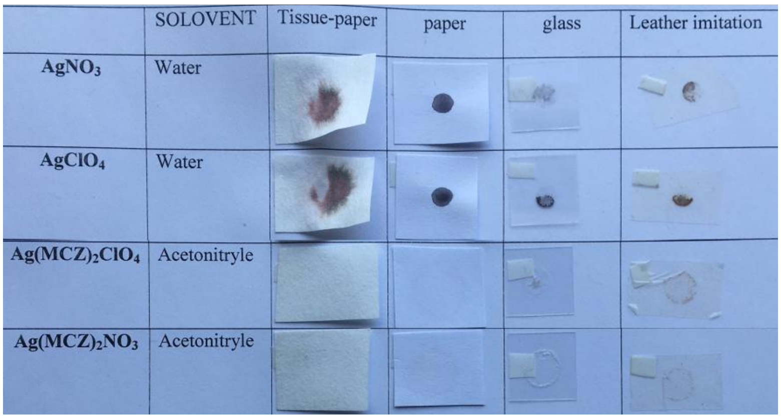

exposition time **40h –light**

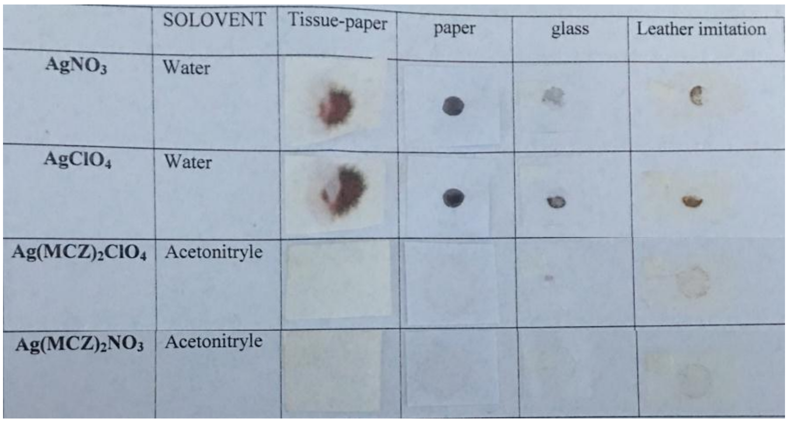

exposition time **48h –light**

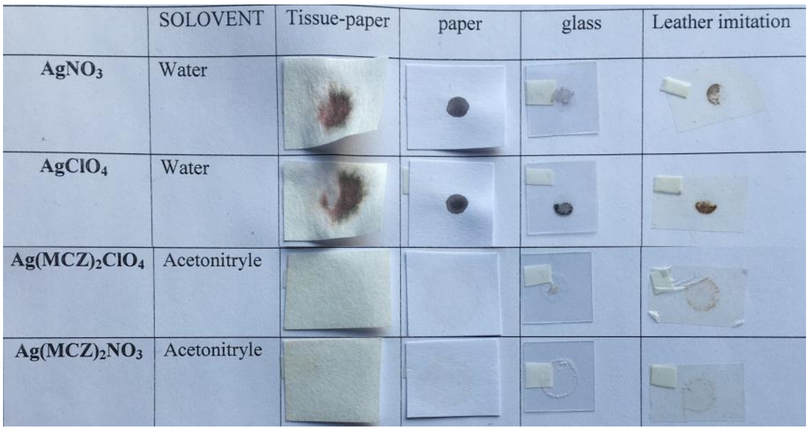

exposition time **52h –light**

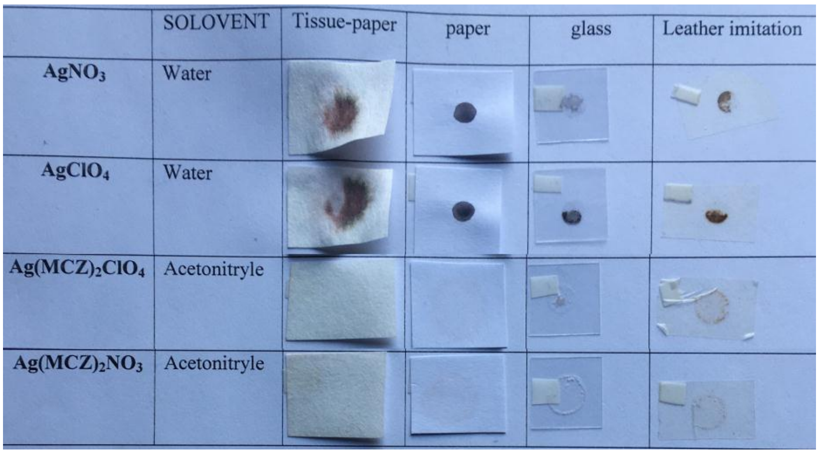

exposition time **60h –light**

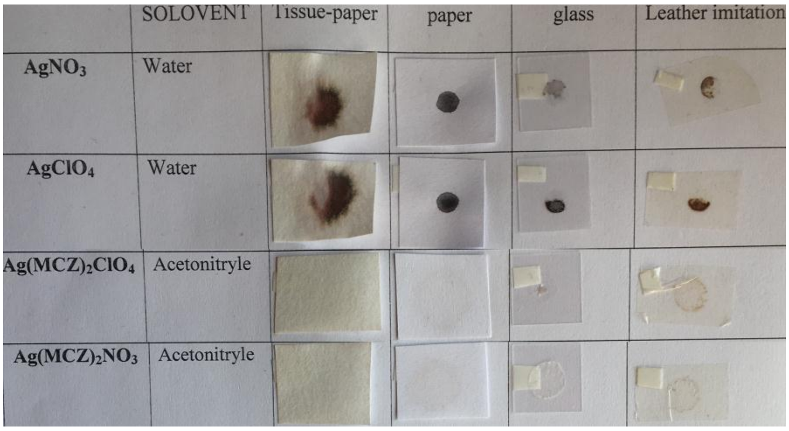

exposition time **84h –light**

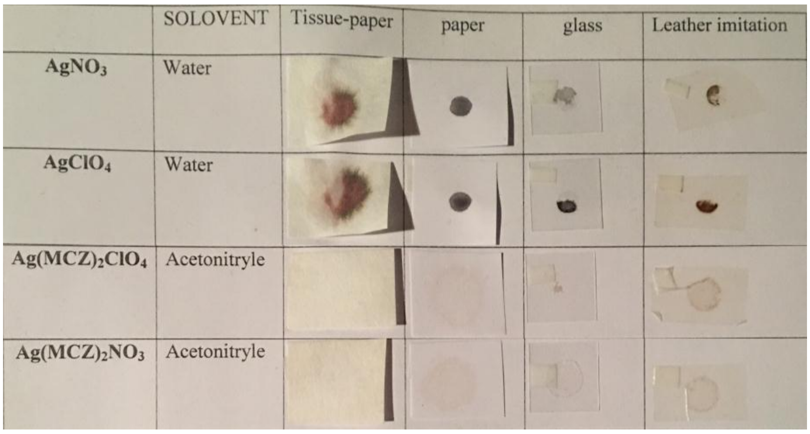

exposition time **108h –light**

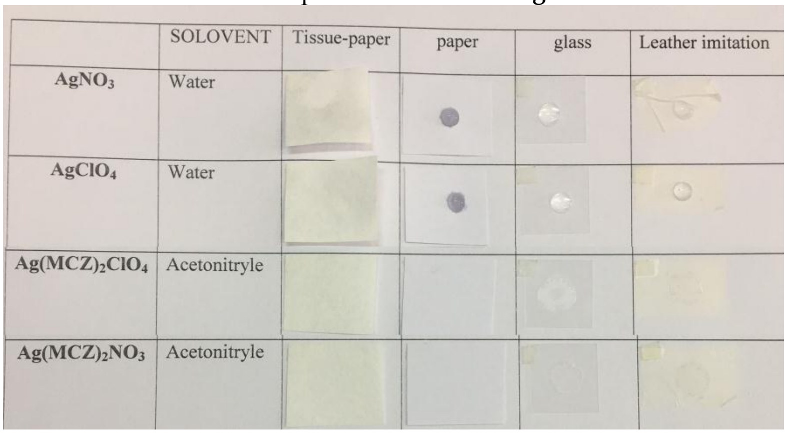

exposition time **0h – dark**

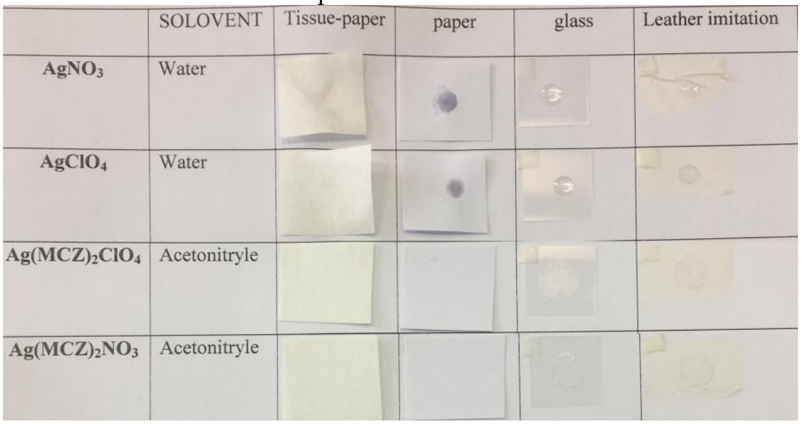

exposition time **1h – dark**

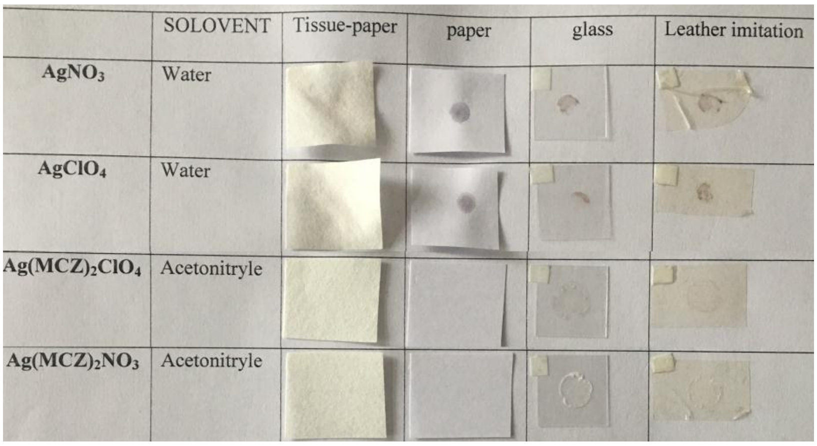

exposition time **4h – dark**

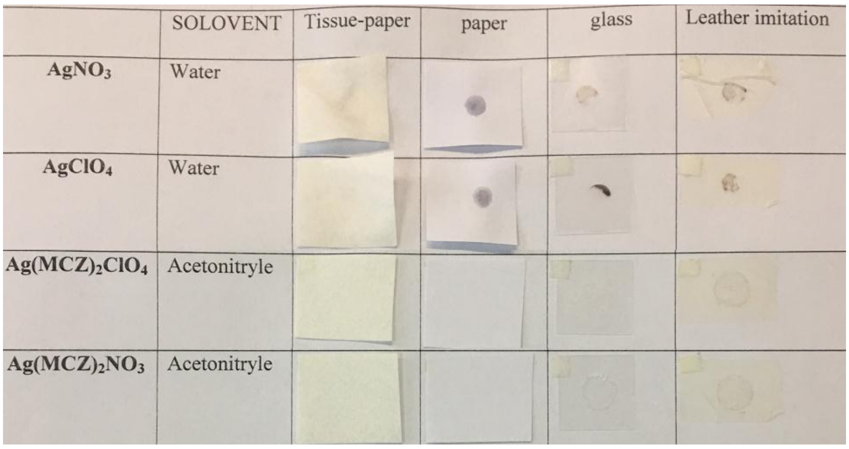

exposition time **18h – dark**

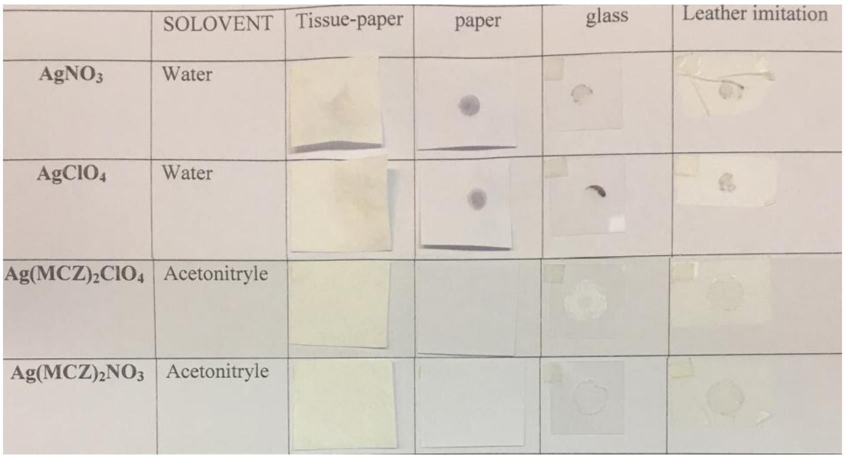

exposition time **24h – dark**

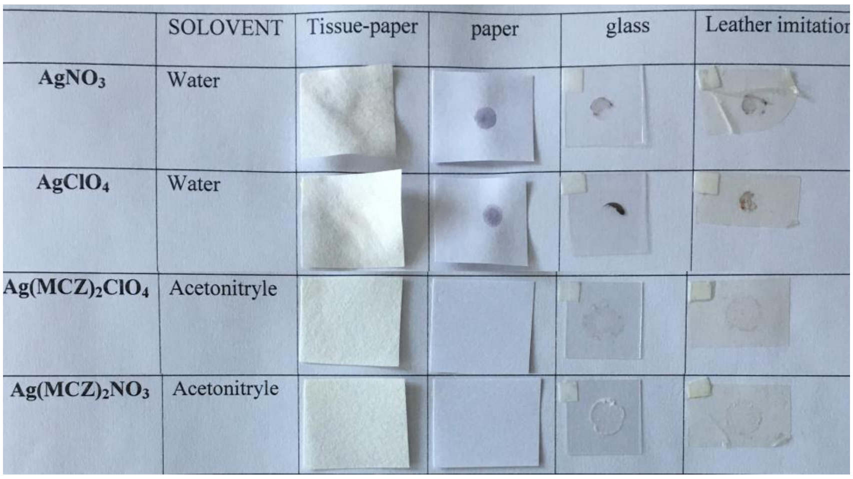

exposition time **40h – dark**

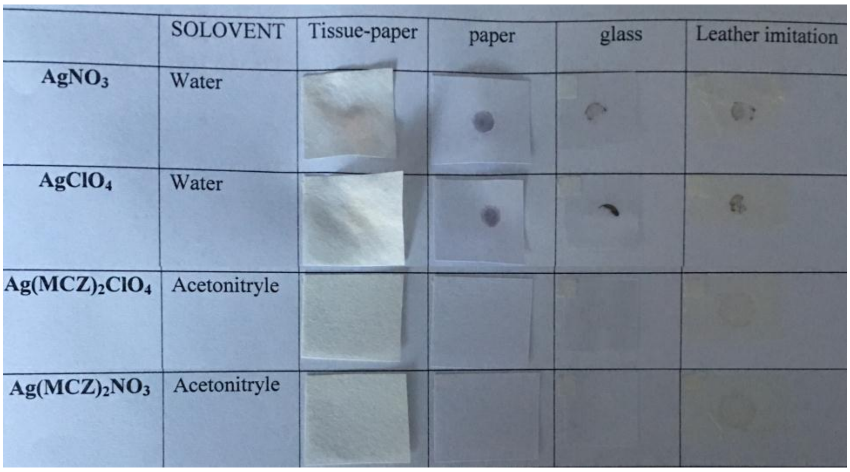

exposition time **48h – dark**

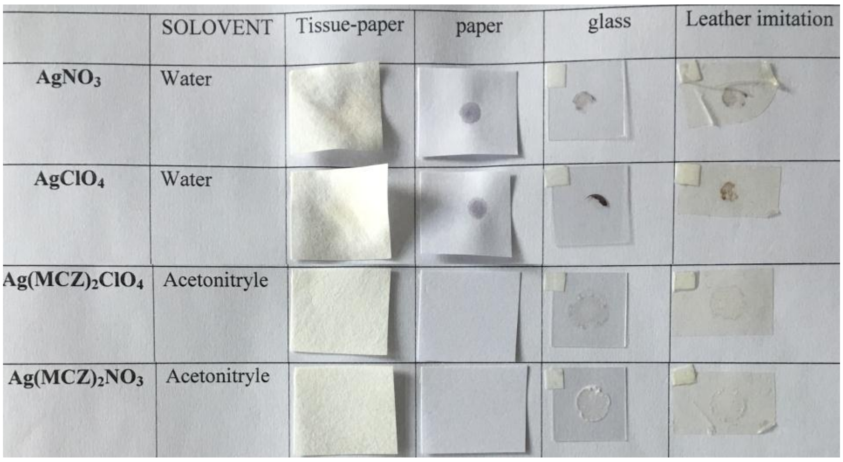

exposition time **52h – dark**

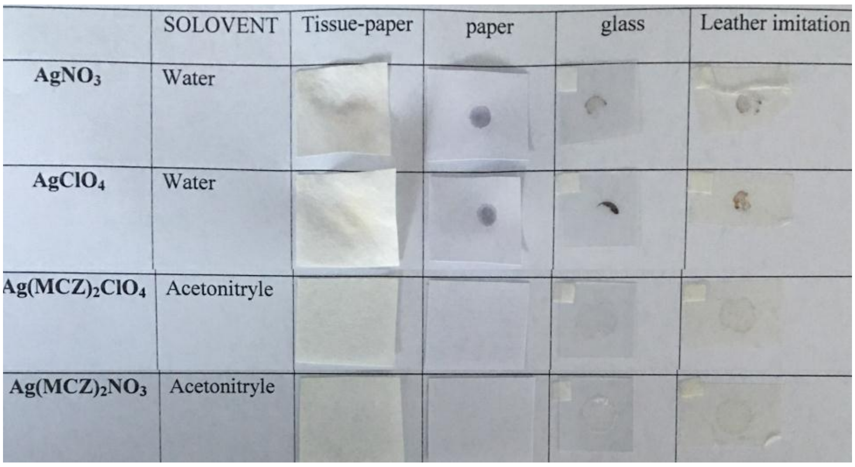

exposition time **60h – dark**

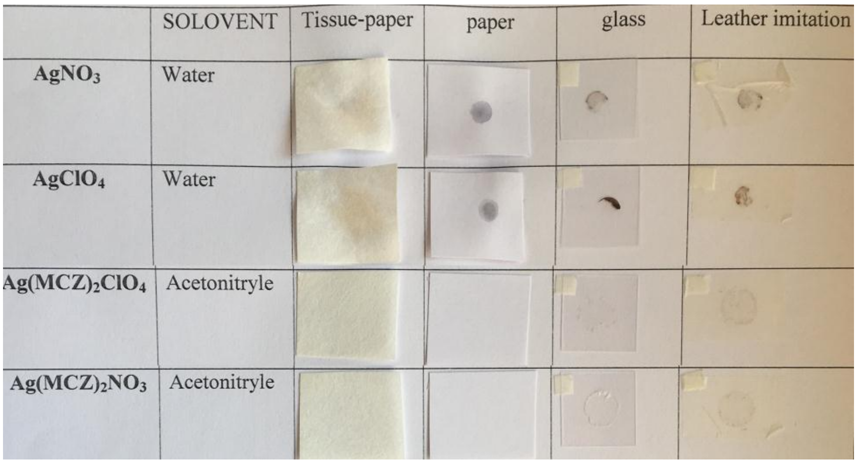

exposition time **84h – dark**

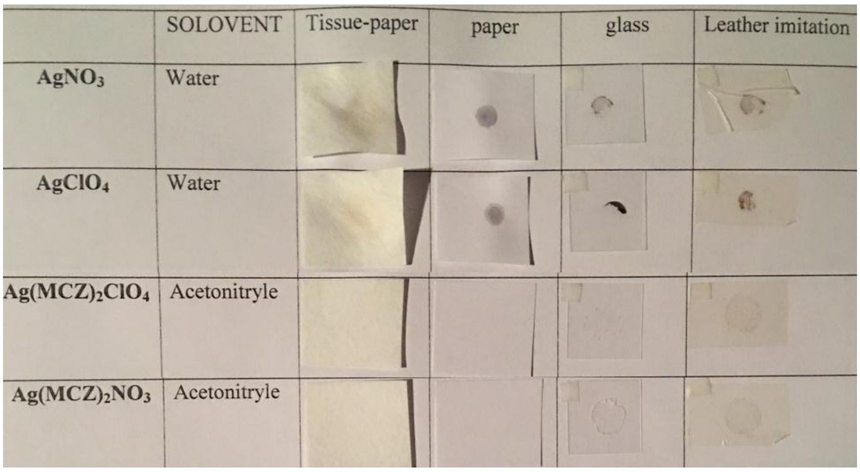

exposition time **108h – dark**

Figure S3. Different substrates (tissue paper, paper, glass, synthetic leather) impregnated with 0.05 mol/l solutions of $AgNO_3$, $AgClO_4$, [Ag(MCZ)$_2$NO$_3$], [Ag(MCZ)$_2$ClO$_4$], exposed to in direct light and dark at room temperature.

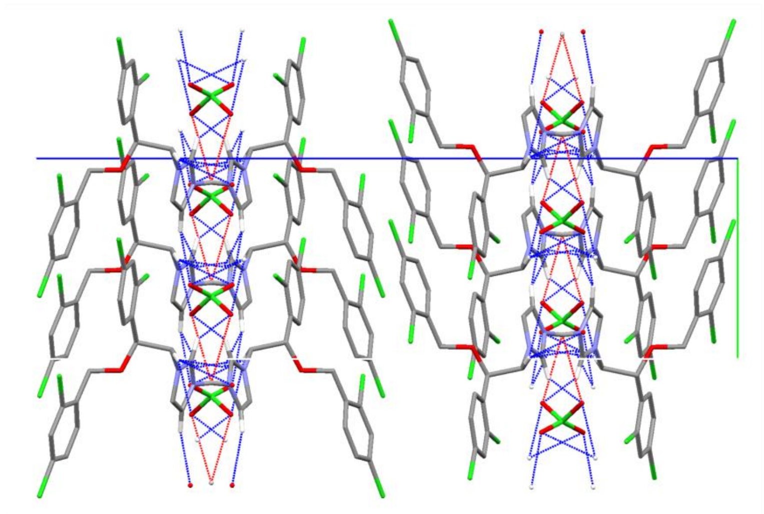

Figure S4. Molecular layers *ab* in the crystal structure of [Ag(MCZ)$_2$ClO$_4$] (**2**).

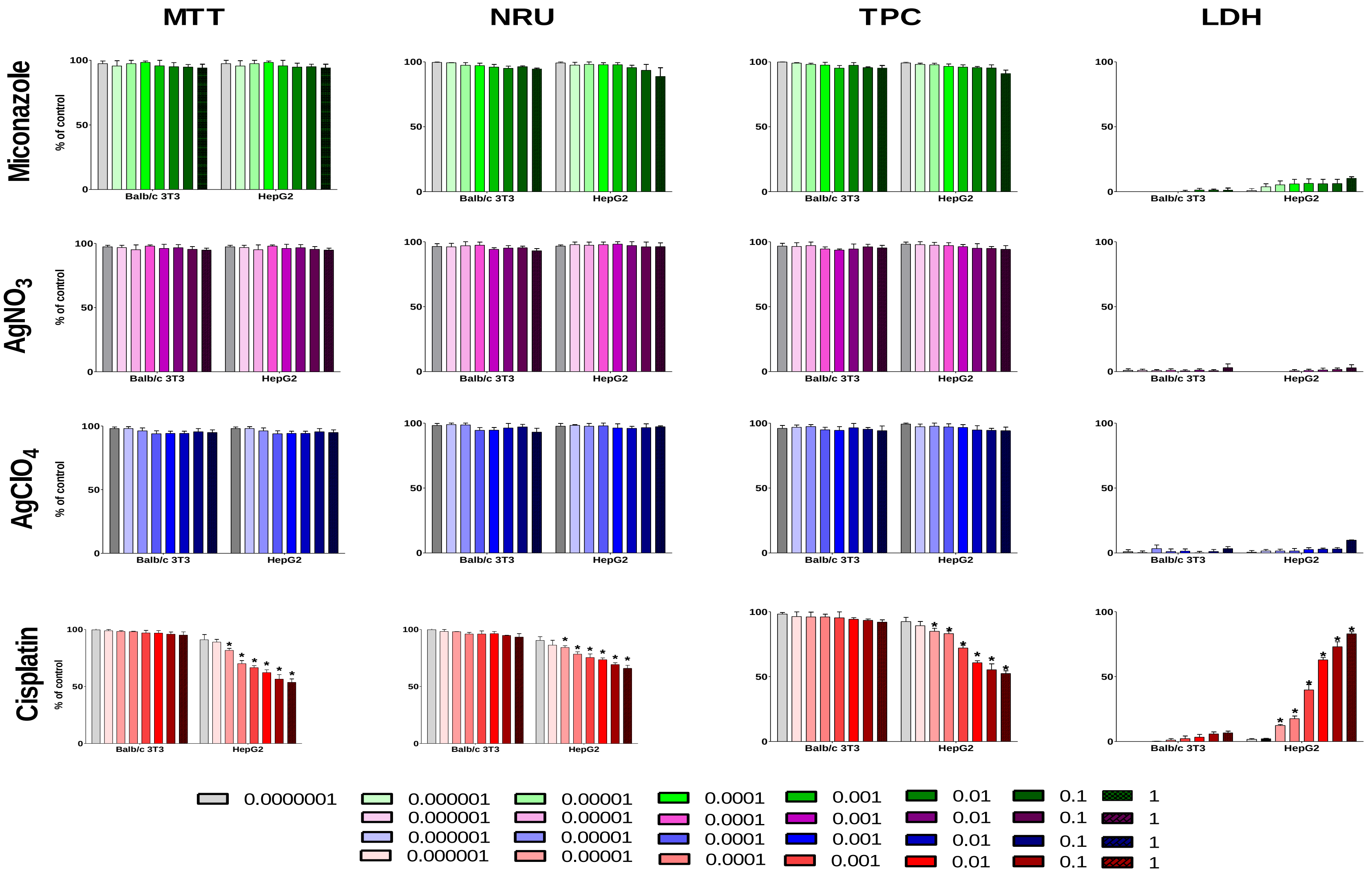

Figure S5. Concentration and cell model-dependent decrease in viability after 72 h exposure to study compounds (miconazole, $AgNO_3$, $AgClO_4$ and cisplatin) assessed by MTT, NRU, TPC and LDH assays. The results are expressed as mean ±SD of three independent experiments. *p ≤0.05 in comparison with control.

Table S1. Aromatic π···π interactions (Å,°) for silver(I) complexes **1** and **2**.

Compound	Interaction	*Cg*(I)···*Cg*(J)	α	Cg(I)perp	*Cg*(J)perp	Slippage
**1**	*Cg*(1)···*Cg*(2)vi	4.106(4)	2.2(4)	3.543(3)	3.546(3)	2.070
	Cg(3)···*Cg*(3)vii	3.947(4)	0.0(3)	3.609(3)	3.609(3)	1.598
**2**	*Cg*(3)···*Cg*(2)v	3.996(2)	12.7(2)	3.139(2)	3.546(2)	1.842
	Cg(3)···*Cg*(3)vi	3.643(2)	0.0(2)	3.373(2)	3.373(2)	1.378

Cg···*Cg* – distance between ring centroids; α - dihedral angle between planes I and J; Cg(I)perp and *Cg*(J)perp - (interplanar spacing) perpendicular distance of *Cg*(I) on ring J and *Cg*(J) on ring I, respectively; slippage - distance between *Cg*(I) and perpendicular projection of *Cg*(J) on ring I. In **1** and **2**: *Cg*(1) – a centre-of-gravity of heterocyclic ring; *Cg*(2) – a centre-of-gravity of dichlorophenyl ring C6-C11; *Cg*(3) - a centre-of-gravity of dichlorophenyl ring C13-C18. Symmetry codes: **1** (vi) ½ -*x*, ½ +*y*, ½ -*z*; (vii) 1-*x*,-1-*y*,1-*z*; **2** (v) *x*,-1+*y*, *z*; (vi) ½ -*x*, ½ -*y*, 1-*z*.

Final Cartesian coordinates (X, Y, Z in Å) for the gas-phase structure of the miconazole Ag(I) complex with NO_3 (**1**).

```
Ag      0.000000    0.000000    3.337185
Cl      0.487737    8.252639    1.203958
Cl      1.086867    9.814093   -3.841514
Cl     -5.010662    5.341026   -1.437991
Cl     -5.361183    0.228304   -2.986553
 O     -0.818532    4.368937   -0.471444
 N      1.139006    3.785106    1.623009
 N      0.704663    1.997789    2.816743
 C      0.000000    5.384549    0.089700
 H     -0.484499    5.783126    0.990811
 C      1.300947    4.686115    0.483865
 H      1.673177    4.116511   -0.370219
 H      2.049368    5.441680    0.731760
 C      0.793676    2.493227    1.596518
 H      0.611457    1.925995    0.692122
 C      0.997118    3.028504    3.672507
 H      1.014482    2.959889    4.753192
 C      1.256434    4.134900    2.958835
 H      1.508476    5.112686    3.350369
 C      0.265990    6.514532   -0.876848
 C      0.600099    7.796007   -0.461810
 C      0.869988    8.833238   -1.372163
 H      1.154636    9.821430   -1.032543
 C      0.757142    8.543908   -2.697553
 C      0.445283    7.305815   -3.147109
 H      0.381158    7.114972   -4.211231
 C      0.208444    6.293872   -2.244785
 H     -0.026466    5.302476   -2.611990
 C     -2.186936    4.760963   -0.627986
 H     -2.250977    5.646199   -1.262444
 H     -2.618839    5.002680    0.344286
 C     -2.945378    3.617428   -1.258971
 C     -4.266551    3.803225   -1.649122
 C     -5.014405    2.769890   -2.183773
 H     -6.049509    2.922903   -2.463125
 C     -4.414852    1.542961   -2.353160
 C     -3.112048    1.326750   -1.993515
 H     -2.654450    0.355714   -2.136980
 C     -2.385460    2.383197   -1.436385
 H     -1.356695    2.220648   -1.139552
 O     -0.398511   -0.753611    5.698723
 O      0.000000    0.000000    7.777924
 N      0.000000    0.000000    6.571345
 O      0.398511    0.753611    5.698723
Cl     -0.487737   -8.252639    1.203958
```

Cl	-1.086867	-9.814093	-3.841514
Cl	5.010662	-5.341026	-1.437991
Cl	5.361183	-0.228304	-2.986553
O	0.818532	-4.368937	-0.471444
N	-1.139006	-3.785106	1.623009
N	-0.704663	-1.997789	2.816743
C	0.000000	-5.384549	0.089700
H	0.484499	-5.783126	0.990811
C	-1.300947	-4.686115	0.483865
H	-1.673177	-4.116511	-0.370219
H	-2.049368	-5.441680	0.731760
C	-0.793676	-2.493227	1.596518
H	-0.611457	-1.925995	0.692122
C	-0.997118	-3.028504	3.672507
H	-1.014482	-2.959889	4.753192
C	-1.256434	-4.134900	2.958835
H	-1.508476	-5.112686	3.350369
C	-0.265990	-6.514532	-0.876848
C	-0.600099	-7.796007	-0.461810
C	-0.869988	-8.833238	-1.372163
H	-1.154636	-9.821430	-1.032543
C	-0.757142	-8.543908	-2.697553
C	-0.445283	-7.305815	-3.147109
H	-0.381158	-7.114972	-4.211231
C	-0.208444	-6.293872	-2.244785
H	0.026466	-5.302476	-2.611990
C	2.186936	-4.760963	-0.627986
H	2.250977	-5.646199	-1.262444
H	2.618839	-5.002680	0.344286
C	2.945378	-3.617428	-1.258971
C	4.266551	-3.803225	-1.649122
C	5.014405	-2.769890	-2.183773
H	6.049509	-2.922903	-2.463125
C	4.414852	-1.542961	-2.353160
C	3.112048	-1.326750	-1.993515
H	2.654450	-0.355714	-2.136980
C	2.385460	-2.383197	-1.436385
H	1.356695	-2.220648	-1.139552

Final Cartesian coordinates (X, Y, Z in Å) for the gas-phase structure of the miconazole Ag(I) complex with ClO_4 (**2**).

Ag	0.000000	0.000000	1.108972
Cl	-0.568221	4.978660	-3.455700
Cl	-2.816763	9.563791	-4.996415
Cl	0.000000	10.146599	0.827940
Cl	1.343893	9.858790	5.940052
O	-0.541197	6.662724	0.578210
N	-1.426643	3.898630	0.548277
N	-0.646479	1.963191	1.184575
C	-0.817371	5.972681	-0.619126
H	0.087058	5.448874	-0.955633
C	-1.903204	4.957113	-0.309529
H	-2.740544	5.463483	0.175152
H	-2.267912	4.525799	-1.244098
C	-1.090160	2.663736	0.165985
H	-1.172490	2.291012	-0.847517
C	-0.691200	2.804924	2.275862
H	-0.385650	2.545274	3.281899
C	-1.172970	3.995049	1.896135
H	-1.331714	4.862088	2.525366
C	-1.304930	6.887588	-1.714688
C	-1.251243	6.512101	-3.042876
C	-1.724926	7.321086	-4.066598
H	-1.673049	7.002763	-5.100459
C	-2.257680	8.529412	-3.728778

C	-2.345036	8.941690	-2.433090
H	-2.773308	9.907591	-2.195375
C	-1.881747	8.114704	-1.422196
H	-1.971013	8.431121	-0.390304
C	0.777413	7.195118	0.605578
H	0.913360	7.906352	-0.210476
H	1.510592	6.394892	0.494364
C	0.953304	7.878689	1.920082
C	0.600288	9.202517	2.117642
C	0.738871	9.830067	3.337214
H	0.472369	10.872731	3.458529
C	1.224932	9.098205	4.394291
C	1.597998	7.784509	4.248900
H	1.992541	7.224591	5.087779
C	1.458448	7.192396	3.007092
H	1.754947	6.158282	2.882251
Cl	0.568221	-4.978660	-3.455700
Cl	2.816763	-9.563791	-4.996415
Cl	0.000000	-10.146599	0.827940
Cl	-1.343893	-9.858790	5.940052
O	0.541197	-6.662724	0.578210
N	1.426643	-3.898630	0.548277
N	0.646479	-1.963191	1.184575
C	0.817371	-5.972681	-0.619126
H	-0.087058	-5.448874	-0.955633
C	1.903204	-4.957113	-0.309529
H	2.740544	-5.463483	0.175152
H	2.267912	-4.525799	-1.244098
C	1.090160	-2.663736	0.165985
H	1.172490	-2.291012	-0.847517
C	0.691200	-2.804924	2.275862
H	0.385650	-2.545274	3.281899
C	1.172970	-3.995049	1.896135
H	1.331714	-4.862088	2.525366
C	1.304930	-6.887588	-1.714688
C	1.251243	-6.512101	-3.042876
C	1.724926	-7.321086	-4.066598
H	1.673049	-7.002763	-5.100459
C	2.257680	-8.529412	-3.728778
C	2.345036	-8.941690	-2.433090
H	2.773308	-9.907591	-2.195375
C	1.881747	-8.114704	-1.422196
H	1.971013	-8.431121	-0.390304
C	-0.777413	-7.195118	0.605578
H	-0.913360	-7.906352	-0.210476
H	-1.510592	-6.394892	0.494364
C	-0.953304	-7.878689	1.920082
C	-0.600288	-9.202517	2.117642
C	-0.738871	-9.830067	3.337214
H	-0.472369	-10.872731	3.458529
C	-1.224932	-9.098205	4.394291
C	-1.597998	-7.784509	4.248900
H	-1.992541	-7.224591	5.087779
C	-1.458448	-7.192396	3.007092
H	-1.754947	-6.158282	2.882251
Cl	0.000000	0.000000	-2.586178
O	1.159352	-0.074955	-1.807909
O	0.004778	1.393453	-3.061691
O	-1.159352	0.074955	-1.807909
O	-0.004778	-1.393453	-3.061691

Final Cartesian coordinates (X, Y, Z in Å) for the gas-phase structure of the metronidazole Ag(I) complex with BF_4^-.

Ag	5.707884	3.826090	1.287703
F	4.900693	3.091445	-1.382909
F	4.863005	2.997695	-3.651020

F	2.989357	2.504363	-2.459524
F	4.826628	1.100430	-2.424342
O	0.212398	4.117303	3.203333
O	1.681822	8.223435	3.926634
O	3.826144	8.534665	4.101040
O	11.524370	2.848550	1.061775
O	10.043902	-0.721517	-0.493539
O	8.052767	-0.875317	-1.340224
N	4.192948	5.219316	1.751896
N	2.188267	6.050970	2.159732
N	2.851214	7.924587	3.676030
N	7.243960	2.403444	1.009253
N	9.134768	1.290166	1.260691
N	8.873516	-0.365414	-0.584743
B	4.365447	2.426160	-2.481366
C	4.348769	6.271149	2.598748
C	3.126425	6.793900	2.854355
C	2.883721	5.096612	1.508128
C	2.306195	4.036773	0.654606
C	0.717091	6.191084	2.165735
C	0.085831	5.522633	3.357731
C	7.264008	1.411008	0.074031
C	8.425839	0.725707	0.217090
C	8.379057	2.312021	1.713213
C	8.742107	3.227742	2.820674
C	10.407504	0.850366	1.870278
C	11.607992	1.434656	1.151813
H	5.288164	6.630047	3.000766
H	1.494686	4.445125	0.076133
H	1.938374	3.236685	1.274683
H	3.065071	3.656888	-0.008511
H	0.311482	5.752951	1.252607
H	0.458638	7.251013	2.171432
H	0.584889	5.843778	4.273203
H	-0.967703	5.797716	3.426115
H	6.489685	1.206520	-0.655008
H	9.154319	2.658704	3.636888
H	7.862850	3.751322	3.156381
H	9.472666	3.938679	2.473139
H	10.464608	-0.238653	1.837754
H	10.430923	1.157875	2.916782
H	12.518398	1.160683	1.686958
H	11.668370	1.012226	0.147727
H	0.024597	3.726647	4.071100
H	11.891269	3.212327	1.882720

Final Cartesian coordinates (X, Y, Z in Å) for the gas-phase structure of the metronidazole Ag(I) complex with CF_3COO^-.

Ag	2.228279	4.825576	14.121951
F	-3.341071	5.923583	14.826505
F	-2.949737	4.500118	13.317023
F	-2.504519	6.600792	13.032169
O	-0.175584	5.123548	13.592227
O	-0.958014	5.377833	15.664248
O	5.015370	1.407532	9.900974
O	1.557121	3.126446	7.811304
O	-0.170526	3.892622	8.899157
O	6.163049	7.211608	17.931227
O	2.511015	6.639300	20.451350
O	0.640897	6.878979	19.359637
N	2.465356	4.478872	11.985680
N	3.220277	3.870048	9.986458
N	1.035992	3.633025	8.807882
N	2.640958	5.185293	16.211621
N	3.695350	5.514603	18.134734
N	1.829498	6.542366	19.428576

C	1.389150	4.308242	11.171376
C	1.835144	3.928474	9.938201
C	3.553987	4.214628	11.249690
C	4.950660	4.295627	11.745773
C	4.170109	3.488952	8.920390
C	4.314989	1.976520	8.799334
C	1.769531	5.799429	17.059291
C	2.404580	6.008567	18.245864
C	3.789919	5.019975	16.885014
C	4.981663	4.314881	16.356668
C	4.796924	5.564397	19.113388
C	5.470844	6.924790	19.136276
C	-1.044344	5.335341	14.434106
C	-2.458792	5.575020	13.848679
H	0.353305	4.450519	11.453638
H	4.944401	4.506497	12.801909
H	5.471478	5.081913	11.225742
H	5.449211	3.357563	11.568538
H	5.145658	3.927057	9.136374
H	3.822330	3.894462	7.969132
H	3.323244	1.526356	8.735490
H	4.849066	1.741389	7.877512
H	0.747893	6.074933	16.828550
H	4.852679	4.129608	15.303593
H	5.103639	3.377742	16.873215
H	5.856236	4.924589	16.509335
H	5.534968	4.800880	18.863161
H	4.405871	5.339641	20.106788
H	4.715762	7.694055	19.304692
H	6.174448	6.959876	19.969337
H	4.367206	1.304098	10.615177
H	6.881607	6.564432	17.855482

Final Cartesian coordinates (X, Y, Z in Å) for the gas-phase structure of the metronidazole Ag(I) complex with $CH_3SO_3^-$.

Ag	0.023692	-0.059977	-0.129788
S	-0.097245	3.360254	0.123943
O	0.898740	2.959421	1.054344
O	-0.315500	2.375185	-0.902015
O	-1.330212	3.767815	0.719767
O	4.582501	-3.654733	1.291560
O	6.567707	0.226774	0.082814
O	5.496781	2.086054	0.063941
O	-4.735190	-3.449819	-1.244682
O	-6.531833	0.249640	0.134448
O	-5.433218	2.091732	0.102049
N	2.165153	-0.236827	-0.394052
N	4.173419	-1.165132	-0.445193
N	5.535452	0.870189	-0.017118
N	-2.103280	-0.261149	0.239071
N	-4.101970	-1.178471	0.435926
N	-5.485729	0.871105	0.161246
C	3.075234	0.736263	-0.175588
C	4.316991	0.183387	-0.203135
C	2.844786	-1.375139	-0.561949
C	2.210893	-2.663756	-0.879016
C	5.213000	-2.212900	-0.545978
C	5.582210	-2.795333	0.795878
C	-3.019842	0.729612	0.131621
C	-4.257030	0.176267	0.246247
C	-2.774305	-1.396161	0.438830
C	-2.135659	-2.698718	0.687770
C	-5.129098	-2.198840	0.718067
C	-5.729310	-2.782805	-0.526766
C	0.546783	4.712779	-0.760468
H	2.851641	1.782266	-0.005963

H	1.143355	-2.533904	-0.937575
H	2.443999	-3.377547	-0.106946
H	2.580772	-3.023472	-1.824386
H	6.105560	-1.787134	-1.006782
H	4.851039	-3.012438	-1.194057
H	6.517719	-3.348635	0.701217
H	5.743873	-1.983642	1.506726
H	-2.805373	1.780258	-0.020148
H	-1.101202	-2.654484	0.391340
H	-2.640064	-3.460273	0.117210
H	-2.198582	-2.934712	1.736711
H	-5.921810	-1.748519	1.317315
H	-4.680736	-3.000473	1.306826
H	-6.525924	-3.479699	-0.262141
H	-6.157399	-1.988105	-1.139524
H	-0.130454	4.978442	-1.554636
H	1.503219	4.446481	-1.177895
H	0.665969	5.551572	-0.095524
H	3.961246	-3.166305	1.854038
H	-5.111878	-3.987310	-1.958902

Final Cartesian coordinates (X, Y, Z in Å) for the gas-phase structure of the metronidazole Ag(I) complex with ClO_4^-.

Ag	2.040775	4.184794	13.595491
Cl	1.353148	7.629108	13.905943
O	2.504972	6.803556	13.623467
O	0.215999	7.111978	13.196766
O	1.618290	8.978298	13.534379
O	1.093595	7.555137	15.338148
O	-0.501412	0.326221	9.711997
O	-0.656965	4.282402	7.623579
O	-0.536211	6.178005	8.684764
O	5.418431	1.307180	17.355403
O	4.189017	4.616566	19.787952
O	3.492146	6.446331	18.834054
N	1.469078	4.033789	11.551347
N	0.930682	3.071586	9.623995
N	-0.285153	4.978002	8.578292
N	2.568762	4.035894	15.649686
N	3.268291	3.127376	17.547976
N	3.662720	5.223744	18.850894
C	0.803263	4.957906	10.813108
C	0.461490	4.377044	9.621822
C	1.544542	2.908141	10.822071
C	2.230232	1.672731	11.274575
C	0.812865	2.043067	8.564983
C	-0.494189	1.272730	8.645652
C	2.810621	5.049772	16.522372
C	3.247303	4.506230	17.698176
C	2.844331	2.887088	16.288787
C	2.678243	1.543544	15.686353
C	3.535517	2.092828	18.562443
C	5.005424	1.738760	18.637407
H	0.581662	5.974666	11.113077
H	2.894554	1.908073	12.088958
H	2.796600	1.257347	10.458112
H	1.497386	0.955432	11.603737
H	1.645508	1.343528	8.652346
H	0.882461	2.525429	7.588886
H	-1.315886	1.976602	8.785865
H	-0.659450	0.750162	7.702311
H	2.679273	6.106092	16.322810
H	2.090443	1.622912	14.787397
H	2.177553	0.895511	16.385847
H	3.645451	1.134326	15.447652
H	2.961856	1.196947	18.320387

H	3.202645	2.452607	19.537141
H	5.586404	2.609937	18.943666
H	5.162249	0.943687	19.367850
H	-0.738565	0.760158	10.546477
H	6.310180	0.933337	17.432384

Final Cartesian coordinates (X, Y, Z in Å) for the gas-phase structure of the metronidazole Ag(I) complex with NO_3^-.

Ag	5.480075	10.440554	5.839319
O	0.788590	12.631893	8.633466
O	2.579952	9.659084	11.684654
O	3.041629	7.890933	10.503904
O	10.175353	12.891623	3.728613
O	8.542249	10.688642	0.017121
O	7.913718	8.749203	0.783273
O	5.884469	7.912160	5.086008
O	4.671416	7.048047	6.670137
O	5.620714	5.763952	5.212216
N	4.553415	10.551741	7.772456
N	3.384026	11.296909	9.508010
N	2.988762	9.113476	10.655329
N	6.450404	10.873140	3.971311
N	7.685969	11.900775	2.435884
N	8.047885	9.974751	0.890624
N	5.395247	6.925744	5.617444
C	4.176902	9.470331	8.509341
C	3.452263	9.909736	9.577997
C	4.055580	11.639092	8.382439
C	4.238994	13.025425	7.893573
C	2.524323	12.195265	10.300190
C	1.060124	12.064057	9.900051
C	6.817033	9.945733	3.047834
C	7.579085	10.558726	2.094742
C	6.991099	12.041491	3.584822
C	6.831502	13.316739	4.328648
C	8.597345	12.909856	1.866389
C	10.025144	12.665621	2.336514
H	4.412515	8.437429	8.284643
H	4.893982	13.557872	8.562467
H	3.284041	13.521931	7.855159
H	4.672079	13.004805	6.907702
H	2.850000	13.233360	10.152211
H	2.632680	11.954457	11.365964
H	0.786980	11.000712	9.882888
H	0.435637	12.558590	10.655735
H	6.550042	8.896290	3.064403
H	5.970564	13.251302	4.972412
H	6.695982	14.125541	3.630505
H	7.711535	13.498829	4.922208
H	8.274509	13.912589	2.176079
H	8.557827	12.862458	0.770125
H	10.308932	11.630213	2.106300
H	10.704388	13.331400	1.787907
H	1.094309	12.022688	7.943317
H	9.898783	12.112278	4.235595
